# Hemp Extract (*Extractum Cannabis*) in the Treatment of Gastrointestinal Distress and Dyspepsia: Historical Insights from Barcelona, Spain

**DOI:** 10.3390/ph17121585

**Published:** 2024-11-25

**Authors:** Kenzi Riboulet-Zemouli, Josep Allué Creus

**Affiliations:** 1 European Union Traditional Cannabis Medicines (EUTC) Research, 08001 Barcelona, Spain; jac@adnconsult.com; 2 Sociedad Clínica de Endocannabinología (SCE), 08015 Barcelona, Spain; 3 Forum Drugs Mediterranean-FAAAT, 75017 Paris, France; 4 Apdena Consult, 08226 Terrassa, Spain

**Keywords:** marijuana, medicinal cannabis, herbal extract, dyspepsia, gastrointestinal distress, gastralgia, stomach disorders, traditional medicine, pharmaceutical history, history of medicine

## Abstract

This study explores the trajectory of interest in and use of *Extractum Cannabis* (hemp extract, i.e., extract of *Cannabis sativa* L.) for the symptomatic treatment of minor gastrointestinal distress and dyspepsia in nineteenth- and early twentieth-century Barcelona (Catalonia, Spain) prior to 1939, through a review of primary sources. The objective of this paper is to present a historical pharmaceutical and applied review of the medical use of the hemp genus (*Cannabis* L.) prior to its prohibition, thereby contributing to its recognition as a medicinal product. The information provided demonstrates evidence of the medicinal use of cannabis within the historical context studied. The interactions between this legacy medical use and the contemporary body of pharmacological and toxicological knowledge (on hemp, its constituents, and the endocannabinoid system in gastrointestinal and stomach disorders) are discussed, providing new possible clinical perspectives. Within its limitations—including the scope, limited accessibility to, and varying quality of archives—this research contributes to a more granular understanding of the historical embeddedness of psychoactive hemp medicines in northeastern Spain, suggesting that medical and pharmaceutical traditions could play a role in informing contemporary approaches to “medical marijuana”.

## 1. Introduction

Currently, there is growing interest in the therapeutic use of *Cannabis sativa* L. (in British English vernacular: “hemp”) in various countries across Europe and beyond. In 2023, the Committee on Herbal Medicinal Products (HMPC) of the European Medicines Agency (EMA) issued a “Call for scientific data for use in HMPC assessment work on *Cannabis sativa* L., flos (Cannabis sativa flowering tops)” and the European Pharmacopoeia is in the process of preparing several monographs on *Cannabis*. These initiatives present a timely opportunity for an applied pharmaceutical historical review, undertaken here with a focus on Barcelona (Catalonia, northeastern Spain).

In the nineteenth century, amidst a backdrop of burgeoning technological, scientific, and commercial progress and challenges in the industrialisation of the kingdom of Spain, medical practitioners and pharmacists in the region of Barcelona embarked on a cautious yet burgeoning exploration of the therapeutic potential of hemp. Although medicinal *Cannabis* had been used for centuries in the Iberian peninsula, this era witnessed a renewed use of hemp—particularly hemp extract (*Extractum Cannabis*)—in the broader healthcare toolkit of the region, albeit for a limited set of indications.

While encouraged by early British and French advances in medicinal hemp research, the pharmaceutical and clinical applications in Barcelona differed, influenced by local empirical observations and partially informed by rich and longstanding familiarity with the *Cannabis* plant. From the mid-1870s to at least 1939, both pure hemp extract and pharmaceutical preparations containing it as an ingredient—administered orally—were indicated in the treatment of various mild conditions, perceptible to laypersons, and generally available without prescription.

The period’s ambivalent perception of hemp—it was viewed as a dangerous intoxicant within “Oriental contexts” and simultaneously as a safe, harmless medicinal commodity in Spain—reflected a complex interplay of pharmaceutical, scientific, cultural, colonial, and legal narratives.

The European Union Traditional *Cannabis* medicines research project (EUTC) reviewed primary sources (administrative, scientific, academic archives, literature, handbooks, etc.) to document the specificities of the traditional use of *Extractum Cannabis* in Barcelona from 1839 to 1939 [1]. The cutoff date, with the rise of Franco’s dictatorship in 1939 represents, an unprecedented historical turning point in Spanish history, including in the local pharmaceutical sector [2], and in the availability of data (Appendix A). This article, presenting part of the EUTC research’s findings, supports and complements earlier studies of the subject [3,4,5,6].

In our article (Section 2), we introduce Spanish medicinal and pharmaceutical hemp history and its singularity, before focusing (Section 3) on the emergence and trajectory of hemp extract’s application in dyspepsia and a variety of mild disorders of the stomach and gastrointestinal tract (termed “gastrointestinal distress and dyspepsia” [GIDD], in this article), one of the preferred therapeutic areas of the time. Section 4 presents the main traditional drug delivery systems identified, highlighting the active ingredient, compounding formulae, and industrial preparations. The social environment in which hemp pharmaceuticals were used is analysed in Section 5, through the regulatory framework and late introduction of prescription requirements, as well as a review of discussions and perceptions around safety. Section 6 discusses—from a contemporary perspective—the historical insights documented, via a brief pharmacological review and considerations of pharmaceutical regulations and policies, particularly in the context of the European Union. After presenting the materials and methods (Section 7) we conclude in Section 8 with thoughts on the implications of our findings for practical pharmacy and future research in Spain and elsewhere, and their relevance to ongoing regulatory discussions in Europe.

## 2. Background: Historical Lacunae and Geographical Imbalance in the Documentation of Medicinal Hemp

The dominance of English in scientific publishing and academia is known to pose some challenges to research and the sharing of knowledge produced in other languages [7,8,9]. This is particularly salient for the history of *Cannabis* L. (hemp) in medicine and pharmacy, which has received scholarly attention predominantly in English-speaking countries and in France, with significant gaps elsewhere, including in Spain and other continental parts of Europe.

In Spain, the documentation of hemp cultivation and its uses remains comparatively limited. With the exception of a few specialised historiographies, which are narrow in scope, Spanish scholarship has frequently drawn on secondary English-language sources to recount the historical development of medicinal hemp within the country’s medical and pharmaceutical context.

Statements such as “The plant and its study was introduced in 1842 by O’Shaughnessy” [10] (p. 56), transposing Anglo-Saxon narratives into Spain, illustrate this bias. Indeed, the contributions of Irish physician William Brooke O’Shaughnessy, although they did spark interest among his English-speaking counterparts to advance the study of hemp pharmacology and therapeutics, are seldom cited in the nineteenth-century Spanish literature (and when cited, the name of this author is always misspelled). Modern-day English-language authors have often exaggerated the extent of O’Shaughnessy’s influence [11]—asserting for instance that he “ [introduced] Cannabis to Modern Western Medicine” [12,13] or to “the European medical community” [14] (p. 195), which seems to have influenced the permeation of these allegations into the description of “medical cannabis” histories [15] in Spain [16], but also in Italy [17] (pp. 34–35) and Latin America [18,19].

Hemp was well-established in the European medical sector since at least the late eighteenth century [20,21] (p. 193) [22] (pp. 108–133), Catalan pharmacy historian Jordi Camarasa García [4] (p. 27) (and others [20] (p. 8346)) have proposed a more modest characterisation of O’Shaughnessy’s contribution: that of strengthening a relatively new clinical and methodological approach to the study of the plant, rather than its “introduction” per se.

Perhaps more importantly, an often-overlooked aspect of O’Shaughnessy’s impact, was his indirect influence on the evolution of hemp from a traditional herbal remedy to a modern, scalable, industrial pharmaceutical product, in the UK:

*“Visiting England in 1842, O’Shaughnessy gave some hashish to a London pharmacist called Peter Squire who owned a chemist’s shop in Oxford Street, requesting that he make a medicinal extract from it. Squire […] patented it as Squire’s Extract and put it on the market as an analgesic”*

[21] (p. 199)

However, again, there is no evidence that Squire’s product was marketed in Spain.

While O’Shaughnessy’s work and Squire’s patent marked a pivotal moment in the understanding of hemp’s therapeutic and market potential among the English-speaking world, it is also essential to recognise its relative and geographically limited scope.

During the same period, in contrast with the limited resonance of O’Shaughnessy and developments occurring in the Anglo-Saxon world, the Spanish medical and pharmaceutical literature on *Cannabis* prominently featured Belgian authors, such as Joseph-François Laneau [Lanneau] (1818–1865), the head pharmacist of Brussels *Hôpital St-Jean*. Laneau’s mentions in primary Spanish sources resemble those for O’Shaughnessy in Anglo-Saxon sources. Surprisingly, Laneau is almost entirely absent from contemporary secondary “medical cannabis history” publications.

Disparities in the historiographies of medical and pharmaceutical hemp use extend beyond leading figures. Many aspects of the non-French and non-Anglo-Saxon history of hemp as a therapeutic agent—market presence, dispensation models, pharmaceutical forms, and therapeutic indications—lack proper scrutiny. This section reviews these historical lacunae, which are necessary for correctly understanding Barcelona’s medicinal hemp history.

### 2.1. Therapeutic Assertions: A Shift Towards Minor Indications

Therapeutic assertions regarding hemp-based medicinal products, particularly in the nineteenth century, were traditionally myriad and varied. Early in that century, French medical practitioners, followed by their British counterparts, held overly optimistic views, envisaging hemp as a potential cure for many prevalent ailments of the era, mainly, communicable diseases. David A. Guba lists “plague, cholera, dysentery, chorea, epilepsy, tetanus, typhus, hydrophobia, migraines, and insanity” as the most-scrutinised indications for hemp in 1830–1850 France [23] (p. 118), roughly similar to the ones documented by James Mills in the British Isles, who also mentions rheumatism and rabies [22]. Rapidly, however, the initial therapeutic claims in these severe conditions were disproven by practice, accelerating the fall into disgrace of hemp remedies in France as early as the mid-nineteenth century [23,24] (p. 473), and in the UK by the 1890s.

The trajectory of hemp medicines within the Spanish medical and pharmaceutical landscape, by contrast, gained momentum during the second half of the nineteenth century. Spain, therefore, renewed its interest in the therapeutic applications of hemp at a time when its efficacy for chronic, contagious, and other severely incapacitating diseases had already been disproven elsewhere in Europe. This delay contributed to shaping a distinct environment for the development of modern hemp medicines in Spain.

Early nineteenth-century French, British, but also Belgian, German, and Italian medical studies reported unsuccessful attempts to treat these conditions. In doing so, however, they also mentioned a number of secondary clinical outcomes, particularly pain relief (including, in some instances, topically), sedation, a general antispasmodic action, and effects on the genitourinary system. These texts, summarised and/or translated into the Castilian (Spanish) and Catalan languages, prompted doctors and pharmacists to focus their interest on the therapeutic potential of hemp to address these secondary outcomes, i.e., for milder health conditions and their symptomatic treatment [1] (pp. 139–154).

Andrés Roig-Traver documented, in a review of mentions of hemp in the Spanish medical literature from 1800 to 1939 [3] (pp. 16–18), a total of 82 therapeutic claims, among which minor afflictions conspicuously prevailed, as evidenced by the disparity between the number of mentions for “insomnia” (*n* = 9), mild GI disorders (*n* = 7; dyspepsia = 4; gastralgia = 2) or “dysmenorrhea” (*n* = 5), and “cholera” (*n* = 2) or “epilepsy” (*n* = 1).

#### 2.1.1. Phases of Development of Modern Medicinal Hemp in Barcelona

The development of medicinal hemp in northeastern Spain can be divided into three distinct phases. During the first phase (1850–1875), the use of hemp for severe diseases was noted, primarily through anecdotal evidence. In 1859, a hemp tincture was included in a Catalan anti-cholera remedy. Although classified as a “venomous drug” under Spanish legislation in 1860, hemp’s therapeutic applications remained largely theoretical, with limited practical use and enforcement [25,26,27,28,29,30,31,32,33,34,35,36,37,38,39,40]. At Barcelona’s Faculty of Medicine, circa 1870, students were still taught that “achiche” (haschish, i.e., hemp extract) “has not become generalised” in medicine [41].

The second phase (1875–1915) marked the peak of interest in medicinal hemp within the Spanish scientific literature [42,43,44,45,46,47,48,49,50,51,52,53,54,55]. During this period, pharmacies in Barcelona and other cities in Spain offered various hemp-based products, such as extracts, tinctures, and syrups, without prescription requirements. The official price lists from Barcelona’s College of Pharmacists and various primary sources reflect the notable availability of these products [56,57,58,59,60].

The third phase (1915–1939) involved the consolidation of medicinal hemp in pharmacies and the beginning of regulatory oversight. As hempseed products disappeared, new forms of hemp extracts were introduced, largely driven by foreign laboratories [61,62,63,64,65]. Despite a decline in mentions within the scientific literature, hemp continued to be taught in Barcelona’s faculties of medicine and pharmacy until the late 1930s [66,67,68,69,70,71,72,73,74,75,76,77,78,79,80,81,82,83,84,85,86,87]. The destruction of public archives in 1939 [88,89] complicates the quantitative analysis of this era, but some records suggest the continued presence of hemp products until the war (Appendix A) [90,91,92,93,94]. Although the scope of the research behind this article stops in 1939, the post-Civil War period likely saw continuity in medicinal hemp use, with Franco’s regime maintaining a relatively lenient approach to Cannabis regulation as reported by others [5,95] (p. 70).

#### 2.1.2. Modern Therapeutic Indications for Hemp Medicines in Barcelona

Evincing a predilection for lower-dose medicinal preparations and for minor ailments over more incapacitating ones, the Spanish medical sector relied, after 1875 and for more than half a century, on hemp extract as a part of its toolkit. This occurred despite the ignorance of hemp’s main active compounds [96]. Until well into the third phase, hemp was dispensed without any requirement for a physician’s prescription. Yet, there is no evidence of any public health concern during the period [97] (p. 88), even though, across the country:

*“Three generics were freely available in any pharmacy at the time: fatty extract (hashish butter), dry extract (hydroalcoholic hashish) or tops (buds), as well as some syrups. Cannabis extract, marijuana, was once extensively used medicinally”*

[6] (p. 23)

The most common indications identified were common menses disorders (dysmenorrhea, metrorrhagia, menorrhagia, and amenorrhea), sleep disorders, dyspepsia, and various forms of mild gastrointestinal distress, as well as general pain relief (particularly to replace opium and other opiates in cases where they are not recommended). Other uses included cough (both via oral and inhaled routes), headaches, urinary retention, and oxytocic applications, as well as topical applications in galactorrhea, and for clavi (corn) and other calli.

These Catalan and Spanish therapeutic indications in part diverge from the most recurrent ones in the English-language literature (1839–1972) identified in 1973 by US psychiatrist Tod Hiro Mikuriya: analgesic–hypnotic, appetite stimulant, antiepileptic-antispasmodic, prophylaxis, and treatment of neuralgias including migraine and trigeminal neuralgia, as an antidepressant–tranquiliser, antiasthmatic, oxytocic, antitussive, topical anaesthetic, as a withdrawal agent for opiates and alcohol dependence, a childbirth analgesic, antibiotic, intraocular hypotension, and hypothermogenic [98].

A note on indications for diseases specific to women: Although the oxytocic indication listed by Mikuriya was also found in Spain, the commonly mentioned use of hemp extract against “hysteria” in the English literature was relatively rare in the Spanish literature, particularly in comparison with dysmenorrhea and other common menses disorders. The supposed use of hemp by Queen Victoria for dysmenorrhea, proven to be a myth [15] (pp. 6–8), [99] is interesting to put in perspective with the prevalence of this particular indication for Spanish women during the same period.

Within this historical, geographically diverse backdrop, insufficiently documented in the literature, this article focuses only on the therapeutic areas of mild GIDD. In doing so, this article endeavours to provide insights into the panorama of the literature and practices related to what we know call “medical cannabis” prior to 1939 in the Barcelona region, attempting a modest contribution to the ethnopharmacology of the area, and to the call for revisited transnational historiographies of traditional herbal medicines under international control—or “new drug history” [100]—while aspiring to advance a more nuanced understanding of the historical therapeutic role of *Cannabis* L., transcending anecdotal narratives.

### 2.2. Names of Cannabis

The way the *Cannabis* plant and its products are perceived and described has evolved notably between the period studied and our time. Discussing the history of medicinal hemp today therefore requires some contextualisation, particularly in terminological terms [101], to prevent anachronistic interpretations. There are two fundamental differences in perception (Appendix B):

(1) Botanical types or varieties: the contemporary dichotomy between the “marijuana”-type and “industrial hemp”-type *Cannabis* did not exist at the time; both types were perceived as the same, single plant: hemp. The *Official Journal of the Spanish State* explained this in 1867:

*“The true hemps of Asia and Europe constitute a single species, cannabis sativa: the differences presented by plants cultivated in India, Persia, and China depend on climatic conditions, and although some botanists have believed them to be sufficient to make of that a different species with the name of Cannabis Indica, this division has not been admitted”*

[102] (p. 4)

(2) Pharmacognosy: The designation of the harvested products did not distinguish the seeds, the leaves, or the “flowers” of the hemp plant, contrary to what is customary today. Harvested parts (hemp tops, which include varying proportions of leaves, seeds, and “flowers”) were often processed, handled, and used together, and named as a single product.

These conceptions of the plant and its products also had an impact on the perception of psychopharmacological activity. To some extent, hemp was almost always expected to display a certain level of psychopharmacological activity. In this article:

- The term “hemp” (British English vernacular for *Cannabis sativa* L., equivalent to Spanish/Catalan vernaculars “cáñamo”/“cànem”—see also Section 7, Appendix B and Appendix C) refers to any plant of the genus “*Cannabis*” and reflects the perception of varietal unicity during the period studied.
- The term “hemp herb” (British English vernacular for *Cannabis herba*) refers to the harvested tops from mature hemp plants (comprising varying proportions of stem, leaves, “flowers”, and eventually seeds).
- The term “dronabinol” is used in the meaning of the international nonproprietary name (INN), referring to delta-9-tetrahydrocannabinol regardless of its source.

## 3. Hemp for Gastrointestinal Distress and Dyspepsia: Emergence and Historical Context

Originating from Asia, the monospecific *Cannabis sativa* genus is considered indigenous to Spain and the broader Iberian Peninsula, where it preceded *Homo sapiens* [103,104]. Humans have harnessed the plant since prehistory, particularly on the Mediterranean coast of the peninsula, for its multiple uses [105,106] (pp. 351–360, 403–407) [107] (pp. 104–106). The oldest archaeological remains of hemp tops date back to 2.900–2.650 BCE [108].

Classical medical treatises from ancient Mediterranean natural philosophers mentioned hemp fruiting tops in various capacities, including as food and sometimes in relation to digestion or stomach disorders [20,106] (pp. 145–147, 163–165, 470, 472) [109,110] (p. 27) [111] (p. 68) [112], at times where food and medicines were not yet perceived as distinct concepts.

On the Iberian Peninsula, the Middle Ages marked a turning point in hemp history. This period, referred to as a whole as “Al-Andalus”, resulted in notable agricultural, medical, and pharmaceutical improvements, which also concerned hemp. The plant was the focus of ethical, theological, terminological, and legal discussions [3] (pp. 18–20) [113,114,115,116,117,118,119,120,121] (p. 366), which later permeated Europe [113,121] (p.163) [122] (pp. 110–24, 267–77, 360–69) [123,124] (p. 31) [125] (pp. 293–301). In Catalonia, the presence of Al-Andalus was limited to the years 717–801; nevertheless, its impact on the history, science, and culture of hemp is as notable as in the rest of the peninsula.

Hemp cultivation and the knowledge of its uses persisted after the progressive Christian conquests of Al-Andalus (the “*Reconquista*”) [103] (p. 266) [126,127] (p. 87) [128,129] (pp. 52, 58). Medicinally, there is evidence of the continued, albeit marginal, use of the different parts of hemp for a series of ailments in the following centuries. However, as with other medicinal plants [130], a gradual loss of knowledge occurred (e.g., with the word “sedenegi”—see detail in Appendix C).

As antique and medieval hemp medicine faded away, new knowledge of the plant was brought into the peninsula by early explorers and commentators of *materia medica* “telling the things” from Africa, Asia, and America [131] (pp. 62–72) [132] (pp. 73–85), such as Diego Garcia d’Orta, Cristobal Acosta, or Nicolás Monardes, who had an important influence on late eighteenth- and early nineteenth-century European scholarship [3].

The development of modern hemp extract pharmaceuticals indicated for GIDD emerged in Barcelona against this rich historical backdrop, and amidst a tumultuous nineteenth century marked by dramatic changes in the professional organisation of the health sector, alongside exponential scientific developments. Surgeons became doctors, apothecaries transitioned into pharmacists, and the role of druggists shifted to the commerce of chemicals, industrial goods, and wholesale [34] (pp. 391–420) [125] (pp. 372–380).

In the 1830s, hemp entered homoeopathic pharmacies [133,134] (p. 545) [1] (pp. 85–86) and began to be discussed in the nascent medical press [135,136] (p. 112) [137] (p. 30) [138,139] and university courses [140,141,142,143,144].

### A True Sedative of the Stomach, Without the Drawbacks of Narcotics

During the “second phase” of the development of the medicinal hemp sector in Spain (see Section 2), as the nineteenth century was coming to an end, specific recommendations for GIDD, with associated preparations and their posology, started to replace general statements on therapeutic areas of usefulness. Given that “[all] branches of medical knowledge […] drank from French sources, through the Spanish versions” [145] (p. 15), it is understandable that the use of hemp for GIDD came from north of the Pyrenees [3] (p. 8), albeit with some delay.

An 1862 French treatise on diseases of the stomach (Spanish translation: 1865) recognised a stomachal antispasmodic and stimulant action in hemp [35] (p. 458). The author introduced a hemp tincture as a stomach tranquiliser dosed at 5 to 10 drops on a lump of sugar, focusing on spasms and cramps particularly associated with muscular irritation [35] (p. 92) and “acute gastric pain” [35] (pp. 98–99); he also highlighted a probable therapeutic usefulness in dyspepsia and other mild GI distress without acidity, although he warned about the novelty of the indication [35] (pp. 295–296). The French stomachal indication was rapidly incorporated by Spanish authors, as in the (modestly titled) *Formulary of Formularies* from 1871, indicating hemp tincture (1 g. for 5 g. 90° alcohol) for stomach aches [43] (p. 624).

Germain Sée (1818–1896), professor emeritus of therapeutics in Paris, specialised in stomach and GI disorders [146], can unquestionably be seen as responsible for the popularisation of the GI-related indications of hemp among the Spanish medical community, where he was highly regarded [145] (pp. 105, 204). His findings were summarised in English in 1890:

“Professor Germain Sée reported an elaborate work as to the value and uses of cannabis indica in the treatment of [gastric intestinal] neuroses and gastric dyspepsia. […] 1. Cannabis should be employed in the form of a fatty extract, in the dose of ¾ of a grain, in three doses daily, in the form of a solution; more than this amount acquires […] symptoms of intoxication. […] 2. It is especially in the non-organic affections of the stomach that cannabis is indicated […]. 3. […] The author shows that cannabis possesses great constancy in its power to arrest painful sensations and restore the appetite, […]. The author shows, however, that cannabis has no action on the gastric contractions or dilatations, although it certainly and distinctly reduces distress, which accompanies these conditions, and which are generally designated under the name of pyrosis; further, gastric digestion is facilitated by cannabis indica, when it is retarded or prevented by loss of nerve power or the excessive pain produced by hyperacidity. Cannabis, as proved by Professor Sée, however, seems to be without any power in producing relief in the various dyspeptic troubles attributable to the amount of acid present.

Finally, cannabis seems to relieve the reflex nervous troubles associated with dyspepsia […]. In conclusion, Sée maintains that cannabis is a true sedative to the stomach, and without any of the inconveniences of the narcotics.” [147] (pp. 684–685).

Sée’s experiences and findings mark a point of inflexion in the discussion of hemp in association with GIDD. In 1900, London hospital doctor Stephen MacKenzie reproduced Sée’s experiments and confirmed his findings. He “only [added] to Mr G. Sée’s conclusions that, beyond purely functional disorders, it has also been favourable in a great number of gastric and intestinal conditions of organic origin.” [148] (p. 152).

The influence of the GI indication is seen in the widely circulated *Formulary of Modern Medicaments* [145] (pp. 33, 36, 41, 86), published regularly by the most reputable Spanish medical journal of the 1890s–1900s, and which focused its section on hemp largely on GI-related disorders, presenting the extract as “a true stomach sedative, without the drawbacks of narcotics, general sedatives, or analgesics” for a variety of mild stomach and GI disorders.

Variants of GIDD indications were often mentioned as the main application for the plant’s extract and tincture, as an influential Catalan pharmacist explained in 1902:

*“above all, it is a very useful gastro-intestinal sedative in cases of stomach cancers and ulcers. Notable successes have been achieved against diarrhea using bismuth subnitrate potions with cannabis tincture instead of laudanum, in strong doses, i.e., 3 to 5 grammes of tincture per day. [...] Germian Sée says that cannabis causes the pain caused by food on stomachs in a state of extreme irritability to disappear immediately. Said sedative action extends over all gastro-intestinal innervations, acting in a much safer way than bromide, and without secondary effects.”*

[149] (p. 334)

Valentín Carulla Margenat, University rector and founder of Barcelona’s *Hospital Clínic*, was keen on teaching his students about hemp’s “only real indication” in GIDD in his lessons on therapeutics [150] (pp. 458) [74] (p. 249) [75] (pp. 464–465), and the faculty of pharmacy teachings often included hemp [1(pp. 113–130)] with some teachers being noted for their well-researched, enthusiastic cannabis contributions [51,52,77,79,82] (pp. 357–359) [78] (pp. 63, 209–210) [79,151] (pp. 96–97, 99–101) [82] (pp. 458–462) [84,152,153,154] (pp. XX, LI–LIII, 125–129).

With the historical trajectory of hemp’s association with GIDD in Spain reviewed, we now shift focus towards an analysis of the specific delivery systems in medical and pharmaceutical practice associated with elements of clinical pharmacology and therapeutics for GIDD.

## 4. Traditional Pharmaceutical Delivery Systems

To understand the traditional pharmaceutical forms and applications of hemp in GIDD in northeastern Spain, a consideration of the multifaceted nature of medicines at the time is critical. The integration of hemp into therapeutic practices, from traditional formulations to standardised pharmaceutical ingredients, signifies an intricate evolution reflective of broader scientific, industrial, and commercial advancements. The late nineteenth and early twentieth centuries marked a pivotal era where empirical methodologies began to influence the standardisation and quality control of medicinal products amidst a constantly evolving environment. Pharmacists, in particular, had to navigate the replacement of “secret remedies” by intellectual property rights, as well as a growing presence of foreign actors on the pharmaceutical market, resulting in both diversifying concurrence and new vulnerabilities associated with increasingly interconnected supply chains [34,155].

The declining presence of traditional hemp extract forms (“haschisch”, “esrar”, “charras”, “guaja”, in particular imported from Mediterranean, Eastern African, and South Asian countries) was paralleled by a progressive replacement by processed, standardised, sometimes proprietary galenical forms. By the turn of the century, at least in name, hemp ingredients were harmonised: pharmacopoeias, wholesalers’ catalogues, and pharmacy jar labels quasi-unanimously used the terms *Herba Cannabis*, *Extractum Cannabis*, and *Tinctura Cannabis* (hemp herb, extract, and tincture). In 1925, these three terms became internationally harmonised as monographs of the Second Brussels Pharmacopoeia Agreement [1] (pp. 77–78) [101] (pp. 13–14), a treaty that prefigured today’s *International Pharmacopoeia* [156] (p. 6) [157] (pp. 55–74):

*“Herba Cannabis Indicae: Flowering and fruiting tops, not deprived of resin, of the female plant cultivated in the East Indies.”*

*“Extractum Cannabis Indicae: Prepare using 90% alcohol by volume.”*

*“Tinctura Cannabis Indicae: Prepare at 10% using 90% alcohol by volume.”*

As discussed, hemp was perceived as a single species, and not only in Spain. In Western medicine and trade circles, the expression “Indian hemp” (*Cannabis Indicae*) served merely as a *pharmaceutical label* to refer to hemp products grown in India or under similar warmer climates (and in regions where seedless tops were made possible by traditional agricultural techniques), thus with an expectedly higher concentration of psychopharmacologically active compounds:

*“We have American, Mexican, African, Indian, etc., cannabis; but these are geographical or commercial terms to designate the country of origin. […] So we have the pharmaceutical term cannabis sativa variety Indica (not botanical) to designate the Indian-grown drug.”*

[158] (p. 410)

### 4.1. Extractum Cannabis as Active Pharmaceutical Ingredient

Hemp’s extract has long been favoured in commerce because of the rapid deterioration of hemp tops after their harvest, particularly the poor long-term preservation of hempseeds (often present in tops). The extract form was also seen as ideal for providing a first step of homogenising, ensuring the increased stability and predictability of effects. Nonetheless, hemp as any herbal extract exhibits variability due to, among other factors, the botanical and agricultural characteristics of crops from different regions; the diversity of cultivation, harvesting, and post-harvesting practices; extraction methods; and age and conservation.

In Spain, the complex exchange of material sourcing that spans various regions (including all around the Mediterranean, West Africa, and up to Crimea [1,37] (p. 255) [126]) challenges attempts of potency assessment. These countries of origin, and the traditional mechanically processed hemp extracts they proposed (often called “resin” or “esrar”, or more often “haschisch”), were progressively replaced as the century was coming to an end and the market shifted towards solvent-based extractions via modern methods and new apparatuses. Variability, however, remained a concern.

The quest to distil plants into their active principles, which characterised the nineteenth century’s scientific pursuits, ignited a competition to identify hemp’s constituents [159] which, nevertheless, remained unsuccessful for more than a century until the identification of cannabidiol (CBD) in 1940 and dronabinol (∆9-tetrahydrocannabinol, THC) in the 1960s [96]. These attempts, although unsuccessful, did generate multiple experimentations of different extraction, purification, and standardisation methods, which contributed to shaping modern European medicinal hemp formulae.

By the late 1840s, the UK and France saw a surge in such research [96]. In France, two pharmacists separately developed quasi-similar extraction methods for hemp [23] (pp. 133–143) (repeated alcohol percolations followed by a water cleaning) that they named “cannabine” and “haschischine”. The two terms stuck as synonyms amongst Spanish doctors and pharmacists [55,160] (p. 77) [161] (pp. 135, 432–433) [162] (pp. 90, 136, 263) [163] (p. 1009)] who made the association between these new forms and “the enervating properties that since Pliny have been attributed to hashisch” [51] (p. 682). Initial claims that cannabine/haschischine was an alkaloid, or a single compound, were rapidly dismissed [51,96,164] (p. 194) [165];; however, while acknowledging the complex chemical composition of the resinous material, it was nonetheless “considered the active ingredient of the plant” [127] (p. 80).

Throughout the period studied, “cannabine”/“haschischine” remained generic names for purified alcoholic hemp extract in the literature [42,47] (p. 316) [48] (p. 157) [57] (p. 27) [162] (pp. 90, 136, 263) [166] (p. 55) [167] (p. 96) [168] (p. 13) [169] (p. 59). In practice, however, “resin of hemp” was also used—like in the Spanish pharmacopoeia’s 1884 monograph—and later *Extractum Cannabis* following the 1925 Brussels Agreement—adopted in the 1930 Spanish pharmacopoeia, which indicated:

*“Exhaust the Indian hemp by leaching, distil or evaporate the alcohol and continue the evaporation, stirring from time to time, especially at the end, until obtaining an extract of soft consistency.”*

[170] (p. 335)

During the early twentieth century, Barcelona’s pharmaceutical community was also keen on preparing fluid extracts of hemp—listed amongst the “most used”—using the *US Pharmacopoeia*’s method: reduce hemp tops into a thick powder, moistened with 300 cc. 94º alcohol, percolated with another 900 cc., reserve, and then “reunite with the extract obtained by the percolation until exhaustion, and form with its mixture 1000 cc. of fluid extract, for every 1000 grammes of vegetable substance used.” [171] (pp. 35, 50–51). Generally available alongside soft or dry extracts, the less-potent fluid extracts were praised for practicality in the compounding of liquid formulations and for use in specific populations, such as “children and weak women against constipation, headaches, insomnia” [172] (p. 395).

#### Assay and Standardisation

In response to the challenges of variability in strength and the presence of adulterants in hemp extracts, methods for assessing their quality and purity were developed. This need arose partly from limitations in chemical research on hemp composition and extraction parameters. The earliest assay methodology reported in the Spanish literature dates back to 1894, relying on 90° alcohol and chloroform solubility, along with ash analysis [61] (p. 318). Over the following decades, advancements in physiological standardisation for hemp extracts marked a significant qualitative leap in the development of modern European hemp medicines, greatly influencing their acceptance by medical professionals [173] (pp. 101–102) [174] (pp. 471–476).

The first hemp extract homogenisation methods were developed in the late 1890s by large pharmaceutical companies present worldwide, including in Barcelona, like Parke, Davis & Co., Ltd. (Detroit, MI, USA, and London, UK), Burroughs-Wellcome (London, UK), and Dausse (Paris, France). These companies played pivotal roles in the standardisation of medicinal hemp products, while simultaneously scaling-up production, distribution, and consequently facilitating access globally.

The products sold by these companies and others to Barcelona pharmacists were generally physiologically standardised extracts, tinctures, and numerous formulae, including soft, dry, fluid extracts of European “Indigenous hemp” [175] (pp. 132, 214), “American hemp” [176], or “Indian hemp” and often designated as hydro-alcoholic, ethereal, fatty, or aqueous extracts [177] (pp. 317,318) [178] (pp. 42–46) [179] (p. 931). Appendix D provides additional background information on these companies and their hemp standardisation methods [180,181,182,183,184,185,186,187,188,189,190].

The transition from traditional resinous forms to more stable, purified extracts thanks to the efforts of companies like Dausse and Parke-Davis, alongside the scientific efforts to standardise the herbal ingredient through physiological assays, heralds the multiplication of pharmaceutical formulations. These ranged from bespoke preparations in pharmacies to, increasingly throughout the twentieth century, standardised, proprietary industrial products.

### 4.2. Compounding Formulae

As an active ingredient, hemp extract was used in a variety of formulae—of traditional origin, and new proposed drug associations. Pure hemp extract, balanced formulations, and formulations incorporating minute amounts of the ingredient, were also present. The last quarter of the 19th century saw an increase in the diffusion of hemp-containing formulae, particularly liquid preparations such as potions and syrups, then the most common pharmaceutical form [43] (p. LX), showcasing the integration of hemp into mainstream medical formulations. There were also a number of hemp pills, appearing as early as 1850, the earliest being the “*Píldoras de Japón*” (Japanese Pills; see Tables below).

During the early twentieth century, local preparations progressively lost ground to industrially produced medicines, particularly foreign ones [155]. In northeastern Spain perhaps even more so, due to coastal port connections. The blend of traditional compounding techniques and emerging industrial processes also led, in some instances, to the registration of locally produced formulae as industrial preparations (see Section 4.3 below).

Among the dozens of recipes [1] (pp. 92–109) [42,43,53,54,61,74,148,161,191,192,193,194,195,196,197,198,199,200,201,202,203,204,205,206,207,208,209], the most recurring compounding formulations directly indicated for GIDD yielded from formularies available to the Barcelona medical readership of the time are detailed in Table 1 (pure hemp extract), Table 2 (the presence of other active ingredients), and Table 3 (broader GI-related indications).

Notably, many of these formulae were derived from French and Belgian recipes, in line with the particular cross-cultural exchange of medical and pharmaceutical knowledge and practices between these two countries and Spain [1] (pp. 101–106) [145]. French formularies also presented GI-related indications as key in medicinal hemp descriptions until the 1910s, such as Astier (“Indian hemp has been recommended [...] mainly as a sedative in gastric pain”) or Martin (“as a local sedative, cannabis is mainly prescribed for stomach pains, where its calming effects are particularly pronounced”—specifying: gastralgia; attacks of mucomembranous colitis, gastric or intestinal pain, palpitations).

The exploration of these formulae highlights the role of hemp extract as an ingredient in formulations for GIDD indications, but also the diversity of pharmaceutical uses and associations. The other ingredients present in some formulae provide insights into the additional challenges that may have shaped the clinical therapeutics of compounded hemp medicine, in addition to the uncertainty surrounding the chemistry, composition, and potency of *Extracta Cannabis*.

### 4.3. Industrial Preparations

The struggle of the late-apothecary/nascent-pharmaceutical sector and druggists—from artisanal, locally prepared, and mostly herbal remedies to the advent of industrial pharmaceutical practices favouring chemical synthesis and scalable production lines—was intense in Spain [34,125,210,211,212,213,214,215,216,217] and particularly in Catalonia [218,219,220,221,222,223,224]. In the case of hemp, in addition to the sociocultural and professional evolution that this shift represented, a certain continuity can be observed between the form and composition of compounded preparations and those of industrial medicines.

Among the types of industrial preparations indicated for GIDD, pills, granules, and tablets, encapsulated in various coatings, gained prominence. Fuelled by the revolution in new compressing and encapsulating machinery [34] (pp. 209, 216–219, 233–257), this form became a predominant medium for hemp extract administration, taking over the liquid preparations, and representing a leap in drug delivery methods for hemp extracts and in discussions of moisture content, conservation, dosing, or the reliability of pharmaceutical effects.

**Figure 1:**
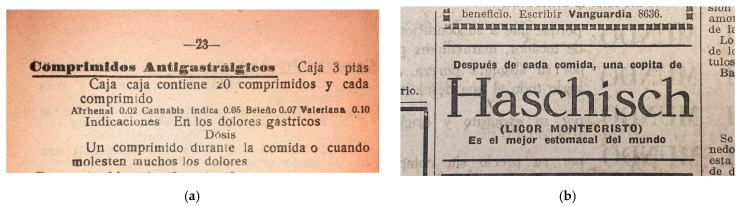
Two of the formulations indicated for GIDD that were present on the market in Barcelona: (**a**) Excerpt from the catalogue of *Laboratorio Farmacéutico Nacional* (1923); courtesy of *Bibliothecas Archivumque ex Legationis Cannabis*; licence: public domain; (**b**) advertisement for the Montecristo Liquor of Haschisch in the Catalan newspaper *La Vanguardia* (1927) No. 46 Vol. 19894, p. 4; courtesy of *Arxiu Històric de la Ciutat de Barcelona (AHCB)*; © AHCB.

**Figure A3:**
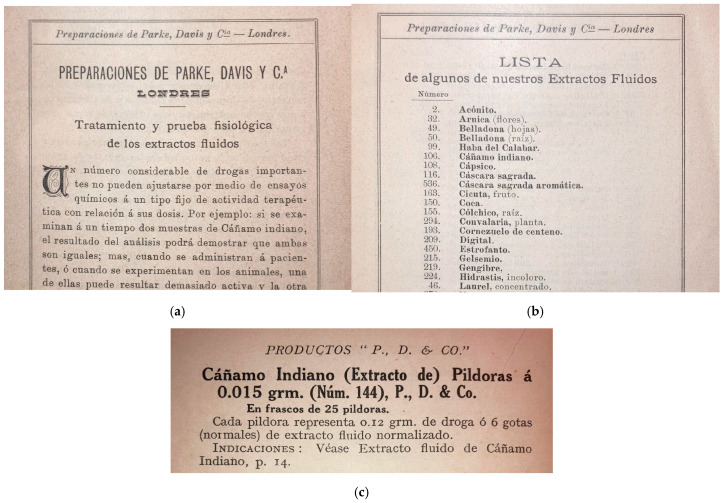
Two Spanish Parke-Davis catalogues. (**a**) Page 1 of the first Spanish catalogue identified, *Preparaciones de Parke, Davis & Co., Londres* (c. 1902) reading: “Preparations of Parke, Davis & Co./London/Treatment and physiological assay of fluid extracts/A considerable amount of important drugs cannot be adjusted via chemical assays to a fixed therapeutic activity related to the dosis. For instance: if one examines two samples of Indian Hemp with some interval of time, the analytical result could show that they are equivalent; yet, once administered to patients or given to animals, one of the two could result in excessive activity, and the other total uselessness”. The demonstration continues by explaining how their physiological standardisation, including of hemp, overcomes these issues. (**b**) Page 3 of the same 1902 catalogue, reading: “List of some of our Fluid Extracts […] 106. Indian hemp”. (**c**) Page 40 of a later catalogue, *Extracto del Catálogo de Parke, Davis & Co., fabricantes de productos farmacéuticos, Londres, Inglaterra* (1920) reading: “Pills of Indian Hemp (Extract of) at 0.015 g. (No. 144), P, D. & Co./In 25-pill jars./Each pill represents 0.12 g. of the drug [herb] or 6 (normal) drops of normalised fluid extract” Courtesy *Bibliothecas Archivumque ex Legationis Cannabis*. Photos by the author. Licence: public domain.

In Barcelona, Parke-Davis started importing pills and tablet triturates of hemp extract in 1888 (catalogue reproduced in Figure A3, Appendix D), followed by others like the French Laboratoire Charles Chanteaud with their “dosimetric granules” of hemp. Local pharmacists also embraced the technology, like Ramón Sol Roigé who patented in 1894 [225] and 1902 [226] processes for gelatine-coated “Pearl-Capsules of Indian hemp” [227,228]. Later on, targeted and multi-compound pills/tablets would appear (Figure 1a; Table 4) [229,230].

Previously favoured in pharmacy compounding, liquid formulations, although relegated to a second rank, remained a trusted delivery method for hemp extract during the transition to proprietary preparations and industrialised methods. For example, in 1910, the “Licor Montecristo de Haschisch” indicated for irritable stomach pain and digestion disorders was patented in València [229], sold in all of northeastern Spain and advertised in high-circulation newspapers (Figure 1b). Names were sometimes changed from “liquor”, “syrup”, or “potion” to “drops”, “solution”, or “liquid preparation”. Table 4 lists some industrial, proprietary medicines marketed in Barcelona for GIDD-related indications [1] (pp. 88–91) [225,229,230].

There were other types of medicines [1] (pp. 88–91), most notably “Bromidia” or the somewhat similar “Chlorodyne”—an infamous hemp-containing chloral hydrate preparation [173,231], first registered as a trade mark in Spain in 1888 [232] and widely marketed for a myriad of indications, including GIDD.

By the end of the period reviewed, new hemp-containing medicines continued to appear, some remaining present on the Spanish market after the civil war (e.g., “Oxigastral” indicated for gastric diseases, chronic gastritis, dyspepsia, and hyperchlorhydria, or “Broluval” indicated, among others, as an “antispasmodic regulator of the neurovegetative system” including for “ [digestive] visceral spasms” as well as “hepatic colic, nephritic colic, vascular spasms, gastric crisis of hyperchlorhydria and ulcer.” [233] (pp. 1226, 1248)).

In addition to these products, both hemp extract and tinctures were also dispensed in raw form (packaged) to patients in pharmacies [54] (p. 56) [58] (p. 46). Progressively, pharmacies turned to dispensing hemp extract in prepared formulations such as pills, tablets, or capsules (either pure or with excipients) instead of the unprepared raw extract. The fluid extract, considered less potent and closer to the tincture, remained available to patients in raw form for longer. In 1917, it was still recommended in “children, 1 to 2 centigrammes per month of age; in older people, 2 to 4 grammes per dose” [172] (p. 395). Hemp tinctures, seen as efficient patient delivery methods, were also dispensed as such, with indications of posology.

## 5. Legal and Social Considerations

### 5.1. Prescription and Legal Requirements (or Lack Thereof)

In an era of significant social, political, and technological change, this diverse and complex pharmaceutical market for medicinal hemp unfolded within, and perhaps owing to, a health regulatory environment marked by disparities between formal legislative frameworks and their practical implementation.

The creation of Spanish pharmaceutical legislation is the result of a long, painful process marked by unceasing controversies, misunderstandings, and conflicting interests between pharmacists, herbalists, druggists, and Spanish authorities. The establishment of “drug control” regulations also spanned decades, often marred by legal ambiguities and regional disparities in enforcement. Throughout the period under scrutiny, access to hemp medicines in pharmacies remained virtually unfettered by health policy requirements such as medical prescriptions until the 1920s.

This situation was associated with an overabundance, rather than a lack, of state regulations. Although it was introduced relatively early compared with neighbouring countries, the Spanish legislative landscape governing pharmaceuticals *and* drug control was marked by a low level of enforcement grounded in opaque, proliferating, and often conflicting laws, ordinances, decrees, and other forms of directives. This regulatory patchwork resulted in an enforcement that was anything but uniform across the Spanish Kingdom and, subsequently, the Spanish Republic.

The 1855 General Health Law and the 1860 Pharmacy Ordinances were the foundational legal structures for the Spanish pharmaceutical sector [34] (p. 39) [215] (pp. 153–154) [217] (p. 31). The Ordinances introduced a detailed division of competences between different actors of the health sector. Fifteen years after France [234] (p. 181) [235] (p. 345) and three years before the UK [236] (p. 593), the Spanish Ordinances introduced a “Catalogue B of venomous substances” (equivalent to modern-day narcotics classification). In contrast to these countries, the first Spanish “Catalogue B” did list “haschisch” alongside substances such as nicotine, iodine, opium, nux vomica… [61] (pp. 324–336) [237] (pp. 309–313) [238] (pp. 17–20). France would only include a *Cannabis*-related entry in 1916 [34] (p. 352) and the UK in 1924 [22] (pp. 188–189). In apparent contrast, in 1866, “cannabine” was included in a decree listing raw drugs authorised for fast-track imports [61] (p. 338).

The practical enforcement of these regulations was another matter entirely. Theoretical restrictions and requirements on sales for pharmacists, herbalists, and druggists, scarcely translated into adherence to the 1860 Ordinances, as evidenced by numerous instances of noncompliance in Barcelona from lax or rogue pharmacists, in addition to an important cohort of

*“practitioners, midwives, somnambulists, quacks and charlatans, legal persons who without pharmaceutical qualifications advertise or sell simple and compound medicines, druggists and herbalists who do not strictly observe [relevant articles of] the Ordinances”*

[125] (p. 378)

By the end of the nineteenth century, noncompliance with pharmaceutical legislation was reaching “practically irreversible proportions in their breadth” [218] (p. 144) when new Pharmacy Ordinances attempted to remedy these issues in 1894. Neither the 1894 Ordinances nor the numerous additional stringent regulations introduced subsequently fundamentally altered the landscape: exceptions, exemptions, and ambiguities continued to provide a large degree of discretion to pharmacists in dispensing almost any medicine other than opiates and cocaine. Pharmacy-compounded formulae were for a long time exempt from most record-keeping mechanisms.

The situation started to change in 1918 with mandatory prescription requirements [239] (pp. 191–192) and actual “drug control”, which included a repression of nonmedical consumption sites and individual possession of “quantities that cannot be justified for medical use through a prescription” [240] (p. 246). Definitive legislation followed in 1919 and 1924 [34] (pp. 365–390) [212] (p. 418), streamlining prescription, creating the first national registry of medicines, and instilling pharmaceutical oversight and reporting. A series of exemptions continued to apply; however, allowing pharmacists to dispense certain *Cannabis* products “subject to the rules of prudence for such cases, under the responsibility of the pharmacist.” [241].

In 1929, under the dictatorship of Miguel Primo de Rivera, a centralised state monopoly (“*Restricción de Estupefacientes*”) was created to oversee and control the pharmaceutical market of the substances listed in the Second Opium Convention [215] (pp. 313–322) [237] (p. 313) [241] (pp. 5–15, 22–23). Mandatory official prescription forms in numbered counterfoil books were introduced, but hemp-containing drugs still “[did] not need to be prescribed by the doctor in an official prescription in order to be dispensed” until 1939 [241] (p. 23).

Important losses in archival materials due to the conflict, censorship, and the generally limited interest in the preservation of pharmaceutical records [242] (p. 39), challenge a proper quantification of the phenomenon in the 1930s, and its demise (see also Appendix A).

This evolving landscape of regulatory leniency and the lax-to-nonexistent prescription and record-keeping requirements (which may have contributed to a lasting presence in pharmacies) especially prior to the 1920s, possibly facilitated the use of hemp medicines for day-to-day conditions such as GIDD. The lax legal environment also underscores the broader societal perceptions of the plant and its safety at the time.

### 5.2. Safety

In addition to this presence on the pharmaceutical market, sources from the period studied suggest a nuanced understanding of the safety profiles of hemp extract and its preparations. While classically associated with so-called “Oriental vices” in the medical literature—relaying exaggerated and biassed, often racist, anecdotes and experiences—the medical uses of the plant made in Spain and Europe were not associated with these “vices”, as authors generally considered that “in Europe it is only used as medication” [243] (p. 89).

Early safety concerns among the Spanish medical community revolved around adulterants present in traditional “haschisch” or “dawamesc” formulations, with doctors and pharmacists complaining about the presence of cantharides, opium, or nux vomica [83] (p. 637) [149] (p. 334), although these concerns ended after the 1890s.

With respect to hemp extract itself, a large consensus was shared among the medical-pharmaceutical community about its safety; although the medical uses on the European continent were always cautiously distinguishing and set apart from the exaggerated non-European “fantasia.” [1] (pp. 158–163) [3] (p. 23) [48,69] (p. 143) [97,149,244] (pp. 768–806). Some authors noted however that “the state of hallucination caused by hashish […] has been greatly exaggerated and disfigured” [28] (p. 83).

Awareness of the potential strength of *Extractum Cannabis* led authors to pay special attention to dosages and routes of administration to leverage its therapeutic benefits while mitigating potential risks. Notably, hemp was generally administered orally, at low medicinal doses, and considered relatively benign in that context [1] (pp. 155–157). Topical, intravenous, intramucosal, or inhaled routes were reported, but less commonly.

Common precautions for use included posology and, in the case of adverse effects, acidic beverages, coffee, and emetics as antidotes. These antidotes were said to efficiently manage adverse reactions, and are routinely mentioned in the literature [1] (p. 158). Some manufacturers even included them in their notice, as for Dr Jimeno’s Syrup: “the inebriation of haschisch dissipates quickly with lemon juice” [245] or Parke-Davis, who recommended “Hot brandy or whiskey; vegetable acids; vinegar, etc.; allow patient to sleep” [246] (p. 271).

However, interactions and contraindications with other ingredients—in formulae and industrial preparations—were rarely accounted for.

By the 1930s, hemp medicines continued to be perceived as safe, as epitomised by a former high-ranking health ministry official, who had pledged “to perform a work of humanity by showing the enormous dangers” of drugs [97]. Although he was advocating fiercely for the prohibition of opium, morphine, and cocaine, he also captured the nuanced feelings of his time about hemp, testifying about Spain in 1932:

*“In medicine, Indian hemp is rarely used as a substance, however, its alcoholic extract and tincture are frequently used. Currently circulating in commerce, under the name of Pure Haschisch, a product prepared by treating the alcoholic extract of Indian hemp with alkali. […] When this pure hashish is administered, at a dose of 0.06 grammes mixed with cocoa or coffee powder, is said to cause a peaceful sleep and a voluptuous inebriation, without unpleasant secondary phenomena.”*

[97] (p. 88)

The consensus on hemp safety appears to have been shared beyond Spanish borders. In 1935, after a complaint of the Egyptian government to the League of Nations against Parke-Davis medicines (see above, Section 4.1), an international survey of public health authorities was conducted. All European countries present (Germany, Italy, The Netherlands, and the UK) declared no evidence of any addiction or public health concern associated with hemp medicines, and the US Surgeon-General commented: “it does not seem that the abuse of galenical preparations of Indian hemp causes any considerable difficulty in the United States” [1] (p. 166–168) [182,247]. Only Canada raised concerns, but about smoked “marijuana cigarettes”, not medicines administered orally. The following year, the League’s experts acknowledged that medicinal *Cannabis* preparations were not a subject of concern in practice, finding that although “in principle and theoretically, preparations containing extract or tincture of Indian hemp could give rise to abuses, […] we do not know of any which has ever produced such effect” [1] (p. 167) [248].

This historical perspective on past perceptions of safety and risk associated with medicinal hemp, in Spain and beyond, contrasts with present-day discussions on an aspect that continues to shape the ongoing debate on the potential role of hemp in therapeutics.

## 6. Discussion

Currently, the safety profile of medicinal hemp products is often conflated with discussions on adult use, particularly the smoked or inhaled forms, diverting attention from the safe profile of orally administered hemp extract. In the first international assessment since the League’s in 1935 [249], WHO experts reported, in 2019, that pharmaceutical preparations for oral use containing ∆9-THC (INN: dronabinol) were still on the market in a number of countries. The experts, however, noted the predominance of synthetically produced dronabinol medicines, reminding:

*“There is no difference between the therapeutic effects or adverse effects of synthetic Δ9-THC and Δ9-THC produced from cannabis plants. These medicines are all taken orally and are approved for use in a number of countries. These Δ9-THC-containing medicines have not been found to be associated with problems of abuse and dependence and they are not diverted for the purpose of nonmedical use. The Committee recognized that such pharmaceutical preparations are formulated in a way that means they are not likely to be abused. Furthermore, there is no evidence of actual abuse or ill-effects to an extent that would justify the current level of control”*

[250] (p. 55)

The alignment of such a modern-day safety assessment of currently marketed synthetic dronabinol invites reconsideration of the equivalent safety considerations on the plant-derived version of the same medicine, ninety years ago.

It is essential to acknowledge the limitations of medical science and pharmaceutical knowledge during the period reviewed, and to refrain from drawing clinical conclusions solely from this extended, nonproblematic use. Nevertheless, the findings presented in this article can benefit from a brief discussion from the standpoint of contemporary knowledge, to understand the underlying motivations of patients and physicians in using hemp for GIDD indications.

Returning to GIDD, in an attempt to grasp further the significance of the findings presented in this article, our discussion commences with a brief examination of the pharmacological profile of hemp and its principal components related to the GI system, followed by a rapid analysis of the pharmacology and toxicity associated with the traditional Spanish hemp formulations documented. The dialogue will then explore some of the social and regulatory implications of our findings.

### 6.1. Hemp Extract as a Modern API

The historical pursuit of hemp extract homogenisation highlights the enduring quest for pharmacological accuracy in dose–response relationship, and a genuine concern for efficacy and predictability in spite of uncertainty and knowledge gaps in hemp’s chemistry. An examination of these historical pharmacognostic practices through a modern lens reveals a close alignment between the different traditional forms of physiologically standardised extracts (soft, dry, liquid extract, tincture) and contemporary definitions in the European Pharmacopoeia’s “Herbal drug extracts—*Plantarum medicinalium extracta*” monograph, particularly as “Quantified extracts”, “adjusted to one or more active markers, the content of which is controlled within a limited, specified range. Adjustments are made by blending batches of the extract.” [251].

Hemp extracts exhibit complex pharmacodynamics, owing to their multi-ingredient composition which extends beyond the well-studied dronabinol and cannabidiol, with other phytocannabinoids working synergistically together as well as with other compounds (termed the “entourage effect” in the case of hemp [252]). For Weiss and Fintelmann,

*“it is a decisive aspect that the plant or parts of a plant which are used for pharmaceutical purposes are regarded as an active substance in its entirety. Herbal medicinal products, in this regard, are always mixtures of a number of substances”*

[253] (p. 1)

In this perspective, physiological harmonisation appears to have represented a way to narrow-down defined pharmacodynamical properties for hemp extracts, thereby managing safety by stabilising outcomes and predictability. From a toxicological and pharmacological perspective, further investigation into orally administered hemp extracts with multiple components would be welcome, particularly due to the putative potential for these components to work together beneficially in mitigating adverse reactions associated with pure dronabinol [254].

### 6.2. Pharmacology

Without aiming for an exhaustive review, this discussion endeavours to bridge historical practices and current clinical and pharmacological knowledge from the perspective of scientific curiosity. It draws upon additional supportive information from recent studies to shed light on the historical use of hemp extracts and tinctures for GIDD (published as this article was in its writing phase) [109].

Based on regulatory approvals and modern clinical criteria of safety, efficacy, and consistency, some medicines based on standardised hemp extract or its components are today generally accepted by the European medical community for a limited number of severe medical conditions. These include the treatment of spasticity in multiple sclerosis, seizures in tuberous sclerosis complex, Dravet and Lennox–Gastaut syndromes, management of anorexia in patients with AIDS-related cachexia, and the alleviation of nausea and vomiting in people treated with chemotherapy. Furthermore, a number of potential other therapeutic areas have been highlighted [250]. Recent research on these different indications and explorations of the endocannabinoid system (ECS) in mammals provides insightful clues as to possible pharmacological perspectives to be drawn from the traditional use of hemp extract in GIDD.

No modern clinical study exists on the safety or efficacy of herbal hemp medicines in the treatment of GIDD as such. Nonetheless, research on the pharmacology of hemp ingredients and their therapeutic applications indicates possible mechanisms of action that could explain the sustained traditional use. Hemp extract-based oral medicines contain phytocannabinoids, including dronabinol (delta-9-tetrahydrocannabinol) and cannabidiol, which interact with the brain and with the GI tract through a mediation of the endocannabinoid neuroreceptors CB1 and CB2 [255] and other signalling pathways [256].

Herbal medicinal hemp products have broad and multitargeted spectra of effects that potentially interact with different symptoms of dyspepsia and GI distress. These include pain relief, anti-inflammatory action, appetite stimulation and antiemetic properties, and indications of anticonvulsant action on GI motility as well as microbiome interactions.

#### 6.2.1. Pain

**Figure 2:**
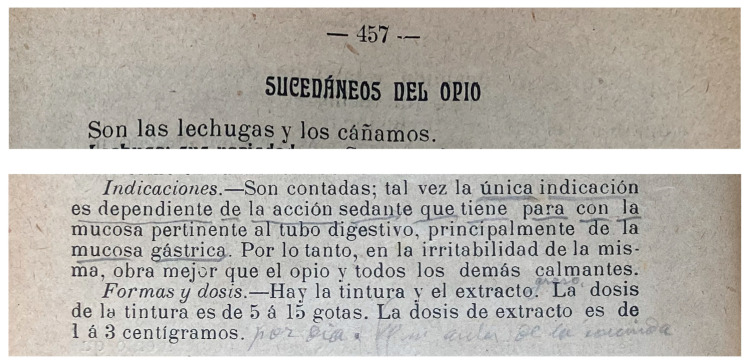
Excerpt from the textbook of Barcelona University rector and professor Valentí Carulla i Margenat (1908), [150] reading: “Opium substitutes./These are the lettuces and the hemps/Indications.––There are few; perhaps the only indication is dependent on the sedative action it has on the mucous membrane of the digestive tract, mainly the gastric mucous membrane. Therefore, in the irritability of the same, it works better than opium and all other painkillers./Forms and doses.—There is tincture and extract. Posology for tincture is 5 to 15 drops. For the extract 1 to 3 centigrams”. Courtesy of *Universitat de Universitat de Barcelona (UB), Arxiu Universitari de la Facultat de Medicina–CRAI Hospital Clínic*. Licence: © UB.

The analgesic effects of hemp extracts are perhaps the most well-known therapeutic application [252]. During the period studied, opium and its active ingredients (e.g., morphine, codeine, or thebaine) remained the most commonly used analgesics, including in the treatment of dyspepsia and GI-related pain. Most opiate and opioid medications modulate GI motility by reducing secretion and alleviating pain, but are also commonly associated with side effects such as constipation, as well as nausea and vomiting. Before the introduction of synthetic analgesics, Spanish doctors (who generally classified hemp amongst “succedaneous of opium”; Figure 2) seem to have favoured hemp extract in GI-related pain precisely to replace opium. As Robert Walton reported in 1937 [257], the comparative advantages of hemp extracts are numerous:

*“[They] do not constipate at all, they more often increase than decrease appetite, they do not particularly depress the respiratory center even in large doses, they rarely or never cause pruritis or cutaneous eruptions and, most importantly, the liability of developing addiction is very much less than with opiates.”*

[98] (p. xviii)

In recent years, promising research has been conducted on the specific analgesic potential of cannabidiol in conditions like inflammatory bowel disease and other functional bowel diseases [258]. Studies suggest that the interaction of phytocannabinoids with the ECS may offer new avenues for treating various functional GI disorders by modulating ECS activity, which plays a significant role in gut physiology and inflammation [252,259].

These differing pharmacological profiles may have played a role in the use of hemp for diagnostics of pain associated with dyspepsia and the GI tract. From a modern perspective, hemp extract could gain new practical clinical utility as an adjunctive therapeutic agent in patients receiving opioid and opiate-based treatments who exhibit negative GI-related outcomes.

#### 6.2.2. Nausea and Vomiting and Orexigenic Effects

The pharmacological properties of hemp components demonstrate significant promise in treating symptoms associated with GIDD. The effectiveness of dronabinol and hemp extracts in mitigating nausea and vomiting, as evidenced in chemotherapy-induced nausea and vomiting management [260,261,262,263,264], and the role of cannabidiol in appetite stimulation [265,266,267] highlights the potential benefits for dyspepsia patients. These findings suggest a therapeutic overlap where the antiemetic and orexigenic effects of these phytocannabinoids could alleviate GIDD symptoms involving discomfort in the upper abdomen and resulting in sensations of nausea, bloating, and early satiety. The regulatory effects of dronabinol and cannabidiol on the endocannabinoid system, which influences GI function [262,268], underscore the interconnectedness of hemp extracts with gastrointestinal health. This synergy points towards the utility of hemp-based therapeutics in managing the complex symptomatology of GIDD through the modulation of neural and hormonal pathways within the GI tract.

#### 6.2.3. Gastrointestinal Motility

Phytocannabinoids have garnered attention as neuromuscular agents for their antispasmodic and anticonvulsant properties, suggesting their potential as therapeutic agents for the management of several seizure disorders [269]. Their interaction with the endocannabinoid system, which modulates neuronal excitability and neurotransmitter release, can exert anticonvulsant effects.

Research has focused particularly on cannabidiol pharmacology, as this substance has anticonvulsant effects through various mechanisms, including the modulation of calcium ion channels, the inhibition of adenosine reuptake, and interactions with serotonin receptors [270]. Cannabidiol has also garnered attention for its anti-inflammatory properties and its ability to modulate GI motility and visceral hypersensitivity, which are key components in the pathophysiology of dyspepsia [271].

Although more focused research is needed, this neuromuscular action, possibly associated with improvement in intestinal motility, could explain the favourable therapeutic outcomes underlying the continued use of hemp extracts in treating symptoms associated with GIDD.

#### 6.2.4. Microbiota

Finally, recent experiments in the burgeoning field of microbiome research and the ECS [272] have revealed the interactions of hemp’s constituents with the gut microbiota in human and animal models [273,274]. Early stages of research indicate that hemp could influence metabolic health through gut microbiota [275,276], unveiling new mechanisms of therapeutic action in the context of GIDD [277].

These findings may shed new light on the historical rationale for the use of hemp in a broad spectrum of GIDD indications in early industrial Spain. Further investigations are needed to determine the role of the interaction of hemp extract with the gut microbiota in GIDD pathology.

#### 6.2.5. Polypharmacology of Traditional Spanish Hemp Medicines

A specific exploration of hemp-containing formulations identified earlier reveals both the breadth of therapeutic intentions and the complexity of safety considerations.

Products such as the *Licor Montecristo de Haschisch* or the *Comprimidos anti-gastrálgicos* underscore attempts to harness the synergistic potential of combined sedative, analgesic, and digestive stimulants to address GI discomfort. The introduction of formulations such as Oxigastral and Broluval at a later date continues this practice, incorporating a broader spectrum of components such as antacids, probably in an attempt to target the acidity and spasms associated with GI distress. These preparations suggest pharmacological strategies aimed not only at triggering symptom relief but also at addressing underlying physiological processes such as acidity regulation and nervous system modulation. The safety and toxicity profiles of some of the additional components, such as belladonna or disodium methylarsenate, reflect evolving pharmaceutical practices that would be deemed unsafe by modern standards. They also allow us to relativise the harms associated with hemp [250] (pp. 37–47, 50–55).

Many of the compounding formulae presented a safer profile—with the exception of chloroform-containing forms and the like. The hemp tinctures, the different formulations for potions and syrups, as well as Bories’ “sweetening tisane”, as well as also the various anti-gastralgia pills and digestive-sedative pills, seem to have been aimed primarily at soothing the digestive tract; addressing symptoms such as gastralgia, general discomfort, and other functional GI disorders through sedative and anti-inflammatory actions; or perhaps affecting motility and secretion. As discussed, current evidence suggests that phytocannabinoids can modulate pain and possibly inflammatory pathways and motility in the GI tract, offering putative theoretical bases for these historical uses.

Other pills target more specific causes of GI discomfort, such as hyperchlorhydria, although current research does not suggest any specific modulating effect of phytocannabinoids on gastric acid secretion, in addition to the functional role of the generic ECS.

The potion formula of Da Veiga, Machado, and Fragoso, which integrates the gastrointestinal regulatory properties of coffee brew with its energising effects to counterbalance the mild sedative action of hemp, provides insight into the synergistic approach to leveraging the pharmacological benefits of both substances. Likewise, the presence of calming suppositories is noteworthy, highlighting an attempt to harness hemp’s systemic actions, with possible relevance in addressing the abdominal pain or discomfort associated with GIDD.

In addition to the obvious limitations of potentially hazardous combinations and unsafe ingredients, Spanish hemp-containing medicines provide valuable insights into past pharmacological practices involving drug combinations and synergistic approaches to the symptomatic treatment of GIDD, in addition to aligning with a number of therapeutic pathways ascertained by contemporary research.

### 6.3. Regulatory Relevance

#### 6.3.1. In the European Union

The European Union (EU), through its regulatory framework [278], aims to facilitate access to traditional herbal medicinal products—that have been traditionally used for at least 30 years, including at least 15 years within the EU itself—focusing on ensuring their safety and efficacy on the basis of traditional use rather than clinical trials.

In January 2023, the EMA’s HMPC, which is in charge of the assessment of herbal medicines, issued a call for scientific data on *Cannabis* [279], reflecting a recognition of hemp’s therapeutic potential and aligning with a broader regulatory openness to the evaluation and potential incorporation of herbal medicines based on well-documented traditional uses and contemporary scientific evidence.

Although the EUTC research project had begun before and independently from that call for contributions [280], our findings directly input HMPC’s efforts by documenting the historical efficacy and safety profiles of hemp extracts in addressing mild GI-related conditions in Spain until less than a century ago. This study facilitates the use of legacy medical and pharmaceutical knowledge as an additional layer to inform modern regulatory standards. HMPC assessment could facilitate the return of hemp extract and hemp-based remedies in European healthcare, empowering physicians and ensuring diverse and complementary treatment options for patients.

#### 6.3.2. Elsewhere

Outside Europe and the EU, the assessment and recognition of traditional herbal medicinal products is more disparate and complex [281]. Nevertheless, the WHO recognises “traditional and complementary medicine” and promotes its incorporation into healthcare systems, including as part of public health strategies of primary care; traditional medicine is defined as:

*“the sum total of the knowledge, skill and practices based on the theories, beliefs and experiences indigenous to different cultures, whether explicable or not, used in the maintenance of health as well as in the prevention, diagnosis, improvement or treatment of physical and mental illness.”*

[281] (p. 8)

If the dominance of English-language literature is problematic in the case of Spain, its impact is exacerbated in the case of indigenous medical knowledge systems [8]. In 2021, a database compiling documented traditional medical uses of hemp across the world presented 324 entries under “digestive system and nutritional disorders”, almost all outside of the European continent [282]. More recently, Thapa and colleagues explored in-depth a number of GI-related uses from Asian ethnomedical knowledge, e.g., powdered dried hemp tops orally administered to treat diarrhoea and abdominal cramps (a study discovered during the finalisation stage of the present article) [109]. These findings strongly corroborate the need for further interest in the application of herbal hemp remedies for a variety of GIDDs and other related disorders.

Finally, the rediscovery of traditional uses of medicinal plants such as hemp can provide critical insights into the development of modern healthcare systems, which are rooted in the sum of human knowledge. This is particularly relevant to developing countries and regions home to indigenous peoples and local community custodians with traditional medical knowledge and biodiversity. In this context, an often overlooked aspect (in exploring traditional knowledge currently in use in these regions of the world and bioprospecting medicinal plant varieties) is the need for a respectful, culturally sensitive approach, fully considering local customary practices and relevant legal provisions against intellectual property-based misappropriation and other forms of biopiracy [283,284] and derived ethical standards [285,286], including full free prior informed consent and access and benefit sharing mechanisms [287,288] (pp. 714–717).

#### 6.3.3. On Bans

The various forms of legal, social, and cultural bans and prohibitions affecting hemp, reflecting a blend of sociopolitical motivations rather than a foundation in scientific inquiry [249] (p. 4) [289], have edged hemp out of mainstream medical discourse [290]. Additionally, when not directly banning academic investigation into its potential therapeutic role, prohibitions constrained research [291]—often by diverting it away at early selection or funding stages [292]. The regulatory stance of prohibition, which emerged prominently in the second half of the twentieth century, and the perception of deviance and stigma that it attached to the plant [293,294,295], invariably lowered the place of hemp in research agendas, across disciplines.

The scarcity of public access to scientific information about hemp has been shown to foster misjudgement and negative attitudes towards that plant and its uses [296]. It could be argued that prohibition, by hindering science, facilitated the negative views of *Cannabis* justifying exceptional measures such as comprehensive bans, in a sort of self-fulfilling prophecy.

In the realm of historical medical and pharmaceutical knowledge, prohibition acted as a deterrent to academic inquiry, contributing to profound lacunae (beyond British and American medicine), leaving large parts of the rich landscape of medicinal hemp history underexplored. Recent scholarly efforts (e.g., Kozma [297,298] or Duvall [15] for African contexts; Mills [22] or Guba [23] for colonial Europe) have begun to challenge some historiographical gaps, illuminating new aspects of hemp’s medical, cultural, and social history, and challenging entrenched myths which at times offer critical, foundational reassessments of established narratives.

This study may contribute to revisiting another long-standing misunderstanding, the erroneous belief that medicinal *Cannabis*, particularly when administered orally, is inevitably associated with public health risks and a liability to provoke dependence. In contrast, decades of use for GIDD outside the realm of prescriptions by people in northeastern Spain without any public outcry or prohibitive scrutiny, challenge the controversies that today tend to capture public attention and scholarly scrutiny.

The rediscovery of such historical evidence invites reflection on the ways in which legal and social frameworks have shaped, and at times limited, our understanding of both hemp’s history, and its potential medical applications.

This discussion holds significance as it underscores the imperative to shift the research focus: rather than persistently probing into the well-trodden terrain of potential harms or hazards—areas already extensively studied—it presses a recalibration of investigative efforts towards the therapeutic potential of hemp. This includes not only its application as a last-resort treatment for severe conditions, but also its utility for everyday health maintenance. This pivot in research direction is crucial for broadening our understanding of the role of hemp and phytocannabinoids in health and medicine, beyond the confines of prohibitions and (resulting) contemporary scepticism.

## 7. Materials and Methods

The research employed a historical, scientific methodology, starting with a review of medical and pharmaceutical contexts specific to Spain, Catalonia, and Barcelona, guided by seminal works of historians of pharmacy and medicine, and other relevant literature. It then expanded to a review of the secondary scholarly literature related to the “history of cannabis” in medicine, supported by in-depth reviews of Catalan and Spanish medical and pharmaceutical journals and books, focusing on the period 1839–1939.

For primary sources, data collection was conducted between November 2022 and June 2023 and in October–November 2023 across 53 archival fonds, as well as online databases (a detailed examination of the methodology and sources can be consulted in the initial report of findings [1]), followed by data analysis, and a complete reporting of findings in December 2023 [1]. The main sources of primary records and the literature were, in Spain: Archivo General de la Administración (Alcalá de Henares), Archivo Histórico Nacional (Madrid), Arxiu Històric & Arxiu Municipal Contemporani (Barcelona), Arxiu Nacional de Catalunya (Sant Cugat del Vallès), Biblioteca de Catalunya (Barcelona), Biblioteca Pública Arús (Barcelona), Oficina Española de Patentes y Marcas (Madrid), Real Academia Nacional de Farmacia (Madrid), Reial Acadèmia de Ciències i Arts de Barcelona, Reial Acadèmia de Medicina de Catalunya (Barcelona), Reial Acadèmia de Farmàcia de Catalunya-Museu Cusí (El Masnou), Universidad Complutense de Madrid, and Universitat Autònoma de Barcelona, Universitat de Barcelona, among others. And abroad: Bentley Historical Library (Ann Arbor), Bibliothèque des Bastions (Geneva), Conseil National de l’Ordre des Pharmaciens (Paris), Library of the World Health Organization (Geneva), Österreichische Nationalbibliothek (Vienna), United Nations Archives at Geneva, Université de Genève (Geneva), Université Paris-Cité (Paris), Wellcome Library Collection (London). Given the extensive range of archives consulted, a variety of methodological approaches were employed to evaluate the significance and credibility of sources prior to their qualitative analysis. This included cross-referencing to validate findings and ensure accuracy.

The research faced significant challenges, in addition to common limitations related to the complexities of historical pharmaceutical research and biases inherent to historical records. The impact of limited data availability on the research findings was notable: the low conservation rate of private archives and the destruction of public ones (mentioned already in context throughout this article) has had a real impact on the ability to assess objective criteria; for instance, those related to the detail of market presence of specific preparations. The variability in the quality and completeness of archival materials, historical formularies, and pharmaceutical records, as well as inconsistencies in terminology, dosages, measuring units, and formulations, required meticulous cross-referencing and a cautious, multidisciplinary approach to data interpretation. A large volume of data representing anecdotal evidence were not used in this article, owing to insufficient corroborating information. The convoluted historical context in which these documents were produced introduced additional layers of complexity.

Other research challenges were specific to the subject matter. The multiple names and heterographs for the hemp plant are difficult to identify not only in database and OCR search queries, but also in physical archives analyses, e.g., using indexes. As an example, “haschisch” was found to be written as aschics, aschisch, ascisc, asisc, assis, atschisch, axis, chascich, hachich, hachisch, haschi, haschich, haschichs, haschicht, haschis, haschischt, haschish, hascisc, hasheesh, hashich, hashih, hashihs, hashish, hatchis, hatschich, hatschish, haxis, haxix, haxixa, and zashih; in addition to some versions with the prefix “al-”). [299]

Finally, difficulties and delays in accessing some fonds, and COVID-19-related issues, extended the data collection period, resulting in a decreased time for analysis and redaction.

Measures have been converted to SI by the authors. The use of INNs is favoured whenever possible.

## 8. Conclusions

This article traced back the use of hemp in nineteenth- and early twentieth-century Barcelona, shedding light on its role in early modern Spanish medical and pharmaceutical practice. This study significantly contributes to the field of pharmaceutical history by providing novel insights into the therapeutic applications of *Cannabis sativa* L. in the Spanish context, underrepresented in prior research, bridging a critical knowledge gap in the historical use of hemp, and positioning it as a key medicinal resource in GIDD.

These findings suggest that, despite important gaps in documentation, *Cannabis sativa* L. was a trusted therapeutic agent for dyspepsia and other mild gastrointestinal complaints. This historical inquiry fills a gap in our understanding of the medical applications of hemp extract, while providing a valuable contribution to the current evaluations of hemp-based medicines by the European Medicines Agency’s HMPC and future work of the European Pharmacopoeia.

The pharmacological properties of phytocannabinoids align with GIDD symptoms, indicating the need for further research and supporting policies to uncover the mechanisms behind the therapeutic benefits of herbal *Cannabis* products in gastrointestinal conditions. The diverse formulations used also highlight hemp extract’s potential as a promising adjunctive therapeutic agent in herbal formulations for the management of GIDD. Historical records support the notion of hemp’s efficacy in managing mild gastrointestinal issues without presenting any significant adverse health events at low doses, enriching the discourse on its use in the existing diverse contemporary medical approaches to that plant.

This applied pharmaceutical historical review offers a practical application for modern pharmaceutical science by providing evidence-based foundations for further research into hemp extracts as part of contemporary therapeutic regimens, potentially supporting the development of new treatments in the field of gastroenterology.

The article also suggests reconnecting with the traditional perception of hemp “buds” and hempseeds as a unified concept, since this view of hemp tops as a traditional nutraceutical could help revisit the food–medicine and seed–phytocannabinoid dichotomies, thus facilitating the exploration of the interplay between diet, GI health, and GIDD treatment. Perhaps this reconnection should begin with precise terminology [101], such as “hemp”, the vernacular British English word for the *Cannabis* plant, and both its tops and seeds.

Acknowledging the importance of regional and cultural nuances in the historical use of medicinal plants such as hemp underscores the value of localised studies within the broader field of medical history and pharmacology. This approach not only enriches our understanding of past medical practices but also offers nuanced perspectives to inform contemporary and future therapeutic uses of traditional herbal remedies.

The study’s historical findings hold relevance for today’s pharmaceutical standards, supporting the potential reintegration of traditional remedies like hemp into regulated modern medical practice. By providing a historical precedent for its safe and effective use, this work paves the way for reconsidering such remedies in current clinical settings, particularly for managing GIDD

Our study highlights hemp extract’s potential as a valuable tool in the therapeutic field of gastrointestinal disorders; its findings advocate for a deeper exploration of its historical use in different regions of the world—beyond the well-documented Anglo-Saxon context—to inform future research and rediscover safe, traditional applications offering “new” options for the improvement of health and the quality of life.

## Figures and Tables

**Table 1:** Pure Hemp Extract Formulations Indicated for GIDD: Inventory from Barcelona’s Formulary Literature (1870–1941).

Formula’s Name or Reference	Inventor	Composition (g.)	Form Posology	Dates Documented	Some Formularies Containing the Formula
**Sedative Pills for the Digestive Tract Mucosa**	Carulla i Margenat	Hemp fatty extract: 0.0033 to 0.01	Pill. One before each meal.	1914	[74] (p. 249)
**Pills Against Gastralgia and Colics**	Herzen	Hemp fatty extract: 0.015	Pill. One per meal.	1914–1941	[1] (pp. 105–109, 145, 155) [191] (pp. 110–112, 194) [192] (pp. 71, 77–78, 163) [193] (pp. 126–127, 233) [194] (p. 149) [195] (p. 466)
**Potion Against Gastralgia**	Dujardin-Beaumetz	Hemp extract: 0.05 Gummy julep: 100	Potion. 3 to 4 large spoons per day during painful crises.	1915–1941	[1] (pp. 103–104, 107–109, 143, 150) [192] (pp. 71, 77–78, 163) [193] (pp. 126–127, 233) [194] (p. 149) [196] (p. 214).
**Potion A**	Astier	Hemp extract: 0.2 Gummy syrup: 40 Julep: q.s. for 125	Potion.	1911	[1] (pp. 105, 143) [197] (p. 381)
**Haschischine Syrup**	Laneau	Simple 35° syrup: 40 Hemp extract: 0.2 Anhydrous alcohol: 20 drops ^1^	Syrup.	1870–1900	[1] (pp. 102–103, 133) [42] (p. 219) [43,53,54] [148,198,199]
**Haschischine Oil**	Laneau	Hemp extract: 0.4 Sweet almond oil: 30	Vegetable oil.	1871–1900	idem
**Hemp Tincture**	-	Varies	Alcoholic tincture.	1870–1941	Appears in all references above [1] (pp. 58–60, 155)

^1^ Another formula was often listed alongside, replacing alcohol with the same quantity of chloroform.

**Table 2:** Hemp Extract-Containing Formulations Indicated for GIDD: Inventory from Barcelona’s Formulary Literature (1887–1927).

Formula’s Name or Reference	Inventor	Composition (g.)	Form Posology	Dates Documented	Some Formularies Containing the Formula
**Sweetening Tisane**	Bories	Hempseeds: 30 Water: q.s. for 375 of decoction. Then, infuse: Syrup of milkweed: 30 Pansy flower: 15	Infusion.	1894	[61] (p. 455)
**Pills** [atonic and painful dyspepsia]	Martin	Hemp fatty extract: 0.015 Henbane leaf extract: 0.03	Pills. One after each meal (max. 3)	1915	[1] (pp. 103–104, 143, 150) [196] (p. 213)
**Pills** [gastric pain caused by hyperchlorydria]	Martin	Coca leaf powder: 0.05 Hemp fatty extract: 0.01 Belladonna powder: 0.01 Morphine hydrochloride: 0.001 Liquorice powder: q.s.	Pills. 1 to 8 per day	1915	idem
**Pills** [gastric disorders in anemics and chlorotics]	Martin	Iron protoxalate: 0.1 Papain: 0.1 Rhubarb extract: 0.03 Hemp fatty extract: 0.025 (up to 0.04) Nux vomica extract: 0.025 (up to 0.04)	Pills. One after each meal (max. 3 per day; max. 2 if using higher doses of nux vomica and hemp)	1915	idem
**Antigastralgic Pills [A]**	Ségard and Laemmer	Phenacetin: 0.05 Acetanilide: 0.05 Hemp extract: 0.02	Pills.	1927	[1] (pp. 105–106, 145, 155) [195] (p. 466)
**Antigastralgic Pills [B]**	Ségard and Laemmer	Camphor: 0.1 Powdered opium: 0.03 Hemp extract: 0.03	Pills.	1927	idem
**Calming Potion**	Berthier	Hemp extract: 0.25 Light coffee infusion: 60 Sugar: q.s.	Potion. 2 or 3 times at night.	1887–1901	[1] (pp. 107–109) [61] (pp. 452–455) [200] (p. 90) [201] (p. 90) [202] (p. 90) [203] (p. 90) [204] (pp. 99–100) [205] (p. 103) [206] (p. 85) [207] (pp. 98–99) [208] (pp. 98–99).
**Haschischine Potion**	Laneau	Distilled water of mint, cinnamon, or apple: 90 Simple syrup: 30 Sugar: 8 Gum arabic: 8 Hemp extract: 2 to 4	Potion.	1871–1900	[1] (pp. 102–103) [43,54] (p. 958) [148,198,199]
**Potion of Indian hemp, Strong**	da Veiga, Machado, and Fragoso	Coffee infusion: 85 Granulated sugar: 15 90° alcohol: 20 drops Hemp extract: 0.5	Potion.	1889–1900	[148] (p. 155) [209]
**Chloroformic Indian hemp Potion** [gastralgia]	Debove	Mint water: 60 Chloroform water: 60 Hemp tincture: 20 drops	Potion. Take spoonfuls.	1900	[148] (p. 155)
**Potion** [gastralgia]	Martin	Orange blossom syrup: 60 90° alcohol: 10 Glycerine: 10 Hemp liquid extract (USP): 1 Distilled lemon balm water: q.s. for 150 c.c.	Potion. 2 to 4 spoonfuls/day	1915	[1] (pp. 103–104, 143, 150) [196]
**Sedative, Antispasmodic, and Analgesic Potion** [gastric or intestinal pain]	Martin	Orange blossom syrup: 40 Thebaic syrup: 40 Sodium bromide: 4 Hemp tincture: 2 Henbane extract: 0.1 Lettuce water: q.s. for 150	Potion. 2 to 6 spoonfuls/day	1915	idem

**Table 3:** Hemp Extract-Containing Formulations for Other GI-related disorders: Inventory from Barcelona’s Formulary Literature (1850–1927).

Formula’s Name or Reference	Inventor	Composition (g.)	Form Posology	Dates Documented	Some Formularies Containing the Formula
**Japanese Pills**	-	Hemp extract: 1 Stramonium extract: 0.03 Amber and Musk: q.s. for pills of 0.2	Pills.	1850–1894	[61] (pp. 452–455) [161] (p. 158)
**Calming Suppositories [A]**	Ségard and Laemmer	Hemp extract: 0.03 Lupulin extract: 0.03 Opium extract: 0.025 Henbane extract: 0.015	Suppository.	1927	[1] (pp. 105–106, 145, 155) [195] (pp. 137–138)
**Calming Suppositories [B]**	Ségard and Laemmer	Camphor monobromide: 0.12 Hemp extract: 0.03 Lupulin extract: 0.05 Henbane extract: 0.02	Suppository.	1927	idem

**Table 4:** Some Industrial Hemp Medicines Present on the Market in Barcelona, Indicated for GIDD.

Trade Name (Translation)	Producing Laboratory (Headquarters)	Composition in Grammes	Form Posology	Dates Documented	Administrative Registry (Type and Number)
**Capsulas-Perlas Dosificadas de Cannabis** (Dosed Pearl-Capsules of Cannabis)	Farmacia Sol Roigé (Barcelona, Spain)	Hemp extract: 0.05	Capsules (gelatin).	1894–1904	Dirección General de Agricultura, Industria y Comercio (Invention Patent No. 15444; Introduction Patent No. 29902) [225]
**Píldoras de Extracto de Cannabis Nº 144** (Pills of Indian Cannabis Extract No. 144)	Parke, Davis & Co., Ltd. (London, UK)	Hemp extract: 0.016	Pills (gelatin).	1902–1936	Dirección General de Sanidad (Dpt. Servicios Farmacéuticos) Registry No. 1002-2 (1921) [230]
**Píldoras de Extracto de Cannabis Nº 145** (Pills of Cannabis Extract)	Parke, Davis & Co., Ltd. (London, UK)	Hemp extract: 0.032	Pills (gelatin).	1902–1936	Dirección General de Sanidad (Dpt. Servicios Farmacéuticos) Registry No. 1002-3 (1921) [230]
**Tabletillas Trituradas de Cáñamo indiano TT Nº 316** (Tablet Triturates of Cannabis Extract)	Parke, Davis & Co., Ltd.	Hemp extract: 0.016 Milk sugar to 0.13	Tablet triturates.	1902–1936	Unknown
**Licor Montecristo de Haschisch** (Montecristo Liquor of Haschisch)	Salvador Costa Gradolí’s pharmacy (Albal, Spain)	Wine alcohol Sugar Hemp extract Sweet calamus Fistula cane Quassia Gentian	Liquor. 1 to 4 cups.	1910–1927	Dirección General de Agricultura, Industria y Comercio (Invention Patent, 48643) [229]
**Comprimidos anti-gastrálgicos** (Anti-Gastralgic Tablets)	Laboratorio Farmacéutico Nacional (Madrid, Spain)	Valerian extract: 0.10 Henbane extract: 0.07 Hemp extract: 0.05 Disodium methyl-arsonate: 0.02	Tablets, box of 20. 1 tablet during meals or during access of pain.	1923–1936	Unknown
**Oxigastral**	Laboratorios Manuel Moya (Málaga, Spain)	Magnesium perhydrol: 3.00 Sodium phosphate: 7.5 Calcium carbonate: 12.00 Hydrated magnesia: 7.35 Sodium bicarbonate: 30.00 Hemp extract: 0.18	Powder.	1936–1946	Dirección General de Sanidad (Registro 4589; Subregistro E.N. 3739) [1] (pp. 88–91)
**Broluval**	Laboratorios DITER (Barcelona, Spain)	Hydroalcoholic vehicle: 67.5 Valerian amyl ether: 15 Calcium bromide: 6 Estronic bromide: 6 Belladonna fluid extract: 2.5 Hemp fluid extract: 2 Phenyl-ethyl-barbituric acid: 1	Drops, 18 c.c. vials. 40 drops in water, 3 times a day.	1939–1953	Dirección General de Sanidad (Subregistro E.N., 5419 & 6269) [1] (pp. 88–91)

**Figure A1:**
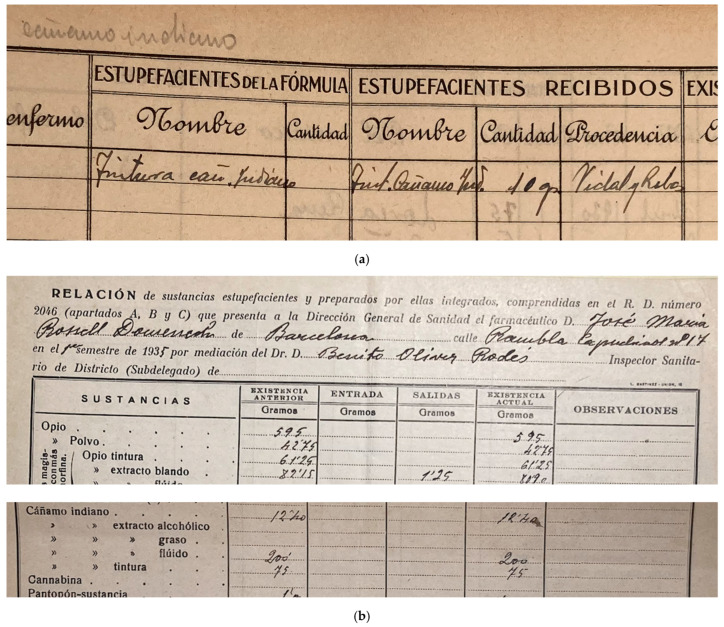
Inventories from Barcelona pharmacies: (**a**) Book of inventory for *Restricción de Estupefacientes* from the Subirá Arumi pharmacy, Barcelona (1929), from the *Col·legi oficial de Farmacèutics de Barcelona* archives at the *Arxiu Nacional de Catalunya* [ANC1-541-UI.201], reading: “Tincture Indian hemp. 10 g. Vidal & Ribas”. (**b**) Reporting form for *Restricción de Estupefacientes* from the Rossell Domenech pharmacy, Barcelona (1st semester 1935) from the same archival fonds [ANC1-541-UI.243 expediente “Estupefaents”], reading: “List of narcotic substances and preparations containing them, included in [Royal Decree] No. 2046 (sections A, B and C) presented to the General Directorate of Health by the pharmacist Mr. José María Rossell Domenech of Barcelona, Rambla de Capuchinos street nº 17 in the 1st semester of 1935 through the mediation of Dr. Benito Oliver Rodés, District Health Inspector (Subdelegate) of the District of …./Substances—Prior Stock (grammes)—Inflow (grammes)—Outflow (grammes)—Current Stock (grammes)—Remarks/…/Indian hemp (12.40)/Indian hemp alcoholic extract/Indian hemp fatty extract/Indian hemp fluid extract (200)/Indian hemp tincture (75)/Cannabine”. Courtesy of the *Arxiu Nacional de Catalunya (ANC)*. Licence: © ANC.

**Figure A2:**
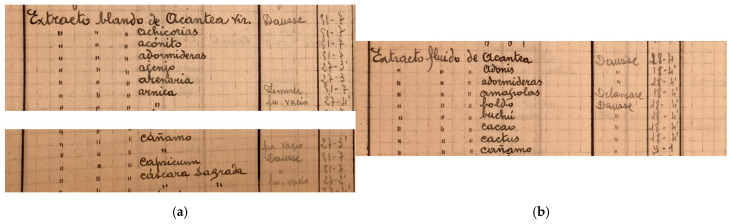
Reproduction of the book of inventory of the pharmacy Serra Millás, Barcelona (c. 1930) from the *Col·legi oficial de Farmacèutics de Barcelona* archives at the *Arxiu Nacional de Catalunya* [ANC1-541-UI.251]: (**a**) The first column lists types of drugs, the second column indicates suppliers, and the third column refers to shelf location; in the first image, “Dausse” is listed as a supplier; in the second image, the first row reads “Fluid extract of hemp” with the second column indicating “jar empty”. (**b**) The last row reads: “Fluid extract of hemp | Dausse | [shelf reference]”. Courtesy of *Arxiu Nacional de Catalunya (ANC)*. Licence: © ANC.

## Data Availability

The datasets presented in this article are available in their respective archival fonds, subject to different access and/or copyright restriction. Part of the dataset is available at cannabistradition.eu/eutc. Requests to access the datasets should be directed to the authors.

## Appendix A. Statistical Data from the Spanish Government (1929–1937)

Hemp entered international drug control in 1925 with the Second International Opium Convention. However, its requirements were very limited, in sharp contrast with the strong controls applied to opium at the time, or with today’s drug control conventions. No prohibition-like measure was contemplated for hemp, nor were any mandatory controls on production or uses. The treaty required only pure hemp extracts and tinctures traded internationally (exported or imported) to be declared, and did not apply to preparations and proprietary medicines:

*“only galenical preparations of Indian Hemp, i.e., extract and tincture of Indian Hemp, fall under the provisions of that Convention. Other preparations of Indian Hemp, even those containing 99 parts or more of Indian hemp extract or Indian hemp tincture to one part or less of any indifferent substance, are not considered as possible agents of drug addiction”*
[300]

The table below corresponds to the data reported to the Permanent Central Opium Board (PCOB), mandated under the Second International Opium Convention for such data collection, vis à vis pure hemp extracts and tinctures traded internationally and related stocks, annually [90,91,92,93,94]. Medicinal preparations that are not pure extracts or tinctures are not included in the figures: only pure extracts and tinctures are reported.

In Table A1 below, the terms “estimates” and “consumption” correspond to trade data between manufacturers and wholesalers, not to the modern understanding of “consumption” by individuals and estimates thereof. Figure A1 shows to an example of a drug control register form from a Barcelona pharmacy.

In addition to the limitation in the scope of what was reported to the PCOB, the figures were incomplete owing to the difficulties in unfolding the regulations and enforcing data collection in an increasingly tumultuous social period. In 1932, Ávila Mata [97] (p. 98) noted that the Spanish law enabling data collection and other drug control provisions had “not produced the desired practical result, mainly because of the abnormal circumstances in which our nation has found itself in the past seven years, which prevented governments from taking the necessary steps to apply the measures adopted”. It is therefore delicate to assess whether the anomalies in reporting figures (1934 and 1935) represent a repeated mistake (unlikely, owing to strict form-filling instructions) or reflect a more accurate reporting of actual quantities in comparison with previous “abnormal” years (surprising by the difference).

**Table A1:** Quantities of “Galenical Preparations” (pure *Cannabis Extractum*, and *Cannabis Tinctura*) and “Indian hemp” (*Cannabis Herba*) as Reported by Spain to the Permanent Central Opium Board (1929–1937).

Year	Product	Estimates for the Year	“Consumption” (by Pharmacists) Reported	Stocks on 31 December
1929	*Galenical preparations*	n/a	n/a	70 or 83 kg.^a^ and 368 kg.^b^ *(PCOB, 1934a: 75,v147)*
1930	*Galenical preparations*	n/a	2 kg.^c,d,e^ *(PCOB, 1934a: 96, 157)*	66 or 72 kg.^a^ and 350 kg.^b^ *(PCOB, 1934a: 72, 131)*
1931	*Indian hemp*	75 kg. *(PCOB, 1934a: 122)*	n/a	n/a
	*Galenical preparations*	5 kg. *(PCOB, 1934a: 122)*	2 kg.^b,e^ *(PCOB, 1934a: 90, 128)*	76 kg.^a^ *(PCOB, 1934a: 69)*
1932	*Indian hemp*	75 kg. (PCOB, 1934a: 112)	n/a	n/a
	*Galenical preparations*	10 kg. *(PCOB, 1934a: 112)*	16 kg. *(PCOB, 1933a: 26, 61; 1933b: 13, 35; 1934a: 48)*	68 kg.^a^ *(PCOB, 1933a: 26, 61; 1933b: 13, 35; 1934a: 41)*
1933	*Indian hemp*	75 kg. *(PCOB, 1933a: 26, 61; 1933b: 13, 35; 1934a: 33; 55)*	n/a	n/a
	*Galenical preparations*	10 kg. *(PCOB, 1933a: 26, 61; 1933b: 13, 35; 1934a: 33; 55)*	41 kg. *(PCOB, 1934b: 2; 1934a: 22)*	98 kg.^a^ *(PCOB, 1934b: 2; 1934a: 7)*
1934	*Indian hemp*	75 kg. *(PCOB, 1934b: 2; 1934a: 29)*	n/a	n/a
	*Galenical preparations*	70 kg.^f^ *(PCOB, 1934b: 2; 1934a: 29)*	26.439 kg.^g^ *GL II* *(PCOB, 1946: 152)*	134.561 kg.^g^ *GL I* *(PCOB, 1946: 163)*
1935	*Indian hemp*	80 kg. (PCOB, 1946: 180)	n/a	n/a
	*Galenical preparations*	75 kg. *(PCOB, 1946: 180)*	14.561 kg.^g^ *L III* *(PCOB, 1946: 124)*	120.000 kg.^g^ *GL I* *(PCOB, 1946: 116)*
1936	*Indian hemp*	85 kg. *(PCOB, 1946: 139)*	n/a	n/a
	*Galenical preparations*	70 kg. *(PCOB, 1946: 139)*	*[Data missing]*	*[Data missing]*
1937	*Galenical preparations*	*[Data missing]*	0 kg.^h^ *(PCOB, 1946: 83)*	12 kg.^i^ *GL III* *(PCOB, 1946: 89)*

Notes on Appendix A’s Table: ^a^ “*Stocks détenus par les négociants en gros*” (stocks held by wholesale traders). ^b^ “*Stocks détenus par l’Etat en vue de la consommation dans le pays pour des besoins autres que les besoins de l’Etat*” (stocks held by the administration for consumption within the country different than the needs of the administration). ^c^ “*Consommation en dehors des besoins de l’État.*” (consumption outside of the needs of the state). ^d^ Comment of the Spanish government: “*Las Necesidades del Estado se cubren por adquisiciones directas de los almacenistas autorizados oficialmente.*” (State needs are covered by direct acquisitions from officially authorised stockists). ^e^ Comment of the Spanish government: “*No existe adquisición por parte del Estado.*” (there is no acquisition by the state). ^f^ Comment of the Spanish government: “*Las cantidades de Cáñamo Indiano importado en años anteriores, se consignaban en la casilla correspondiente, como tal extracto. En el presente por indicar el formulario B.(G) en su nota 3 la cantidad de planta equivalente a un kilogramo, se expresa en planta.*” (Quantities of Indian Hemp imported in previous years were expressed in the corresponding box, as extract. This year, because form B.(G) indicates in its note 3 the amount of plant equivalent to one kilogramme, they are expressed in plant equivalent). ^g^ The difference in these figures compared to other years is surprising. It may be an error, but could also reflect the actual numbers. Indeed, the forms filled by Spanish officials were very clear and carefully worded to indicate how data need to be reported, expressly requesting that no quantities below kilogrammes should be reported (Omit quantities less than one kilogramme from the figures entered in this report. Enter the figures without dots or commas; “*Omettre des chiffres inscrits dans le présent rapport les quantités inférieures à un kilogramme. Inscrire les chiffres sans points ni virgules.*”). In addition, a comparison of the reporting from other fields for hemp, and for other drugs, seems to rule out the possibility of an error. In consequence, for example, the figure “14.561 kg”, which corresponds to the quantity of Galenical preparations reported as consumed in 1935, should be read as *fourteen and a half tons*, and not as *fourteen and a half kilos*. ^h^ Comment of the Spanish government: “*Por las condiciones en que hubo de hacerse la distribución de sustancias en parte del año 1937 a causa de las circunstancias excepcionales de España, algunas de las cifras consignadas han de estar sometidas a ulterior rectificación.*” (Due to the conditions under which the supply of substances had to be made during part of the year 1937 because of exceptional circumstances in Spain, some of the figures recorded have to be subject to further rectification.) ^i^ Comment of the Spanish government: “*No hay más stock que el del Estado. En las cifras consignadas reflejan las exactas en existencias de productos, ya que las que quedan en poder de los Almacenistas son insignificantes a los efectos de esta declaración. Este stock se destina a cubrir todas las atenciones sanitarias del país, tanto de orden público civil como militar.*” (There is no more stock than that of the State. Consigned figures reflect the exact stocks of products, since those that remain in the hands of stockists are insignificant for the purposes of this statement. This stock is intended to cover all healthcare in the country, for both of public civil and military purposes).

## Appendix B. Hemp and Marijuana—Anachronistic Dichotomies for *Cannabis sativa* L.

To contextualise medicinal hemp in the period studied, avoiding anachronisms, it is helpful to briefly remind readers of two important differences between the perceptions of the plant at that time and the present. First, the plant was viewed as a single type (distinct from the current perception of a varietal dichotomy based on psychopharmacological activity, i.e., hemp vs. marijuana). Second, all parts of hemp tops were perceived together as a whole, single product (as opposed to what are currently recognised as distinct parts: “flowers” and “hempseeds”).

With respect to the first perception, what we currently call “industrial hemp” and “marijuana” were not distinguished in terms of their psychopharmacological properties throughout most Spanish history. From antiquity to the start of the Civil War, the *Cannabis* plant was understood as being both “hemp” and “marijuana” simultaneously [301]. It is only in recent decades that an essentialisation of the more-psychopharmacologically active hemp samples vis à vis less-active ones became common ground, as it remains today [302]. Guba eloquently described how this evolution in perceptions was critically influenced by French imperial narratives in the eighteenth and nineteenth centuries, instilling a culturally biassed polytypic concept which nowadays dominates “Western scientific, popular, and legal perceptions of the plant and its byproducts” leading “[scientists], scholars, and laypeople alike [to generally] believe that there are distinct species of cannabis.” [23] (p. 44). Contemporary botanical taxonomy and systematics, however, support the ancient view of a monospecific genus, showing that genetic variations in hemp take place at a low systematic level, and in a much more complex way than dichotomically [303].

In the field, more than the genetic type (in particular, for traditional plant varieties), it is the influence of changing environmental factors—mainly varietal selection, climate, and sowing density—that determines the output of hemp crops, particularly their phytocannabinoid content (responsible for psychopharmacological activity), as Spanish farmers knew:

*“Depending on its destination, hemp is sown more or less densely; if it is to harvest abundant seed it must be sown very lightly, if on the other hand it is to benefit its textile parts it is sown coarsely”*
[304] (p. 4)

The second historical difference in perception concerns the unicity of hemp tops, which were seen as an agricultural product essentially comprising both the seeds and the psychopharmacologically active vegetal parts. This perceived sameness is critical to understanding the mindset of the time.

Phytocannabinoids are contained in epidermal glands (trichomes) covering the pericarp of the plant’s mature gynoecia, which are grouped in dense, agglomerated tops. These glands are present in both specimens with seed-bearing tops, and in tops deprived of seeds (generally called “flowering tops”). The latter tend to have higher yields of phytocannabinoids. In current terminology, “hempseed” refers to the seed kernels from seeded tops, after having been separated from the pericarp surrounding them [101] (the seed kernel itself does not contain phytocannabinoids). Historically, however, such a clear separation did not exist; the term “hempseed” encompassed both the seed kernels and the entire seeded top (i.e., not only the kernel but also the pericarp surrounding it, and its trichomes).

The harvest of seedless tops was not documented in Spain until the very end of the nineteenth century. This is perhaps due to the agricultural requirements for such an outcome, including the need to withdraw staminate individuals from dioecious fields prior to early flowering in order to induce the development of seedless tops. Traditionally, European farmers were not as familiar with these techniques as their counterparts in subtropical regions. As a result, hemp tops in Europe generally contained both seeds and “flowers”.

Recently, it has been proposed that the morphological botanical phenomenon known as “parthenocarpy” might explain the development of hemp plants in flowering and fruiting stages [101,305] (p. 9, App. II), which could shed light on historical seed–top conflations commonly found in primary sources prior to the twentieth century, and this perceived unicity of hemp tops and hempseeds [306].

Irrespective of the cause, “hempseeds” and “hemp flowers” were routinely considered, discussed, and sold together, and viewed interchangeably. This may explain why, rather than the term “flower”, the words “herb” or “leaves” were commonly used to refer to hemp tops with fewer seeds and more phytocannabinoids [307] (p. 394).

The task of separating seed kernels from their pericarp is labour intensive. It is not documented in archives. Instead, several (mostly French) authors depicted the processing of entire tops (with pericarp and leaves) as the traditional postharvest method for hempseed production. In 1807, Lemery explained (in a book published in Castillan several times thereafter) that hemp crops were processed by separating harvests into stems on one side (for fibre production), and “seed and leaves” on the other side [308] (p. 329). In 1843, Lemaire specified two postharvest processing methods, both using the kernel and pericarp, which resulted in a final material with some phytocannabinoid content:

- “the female hemp is uprooted and the seed is harvested by threshing it;”
- “the tops of the hemp [are submitted] to the mortar” [309] (p. 388).

During the period studied, imported hemp tops from colonial regions became preferred for their superior psychopharmacological activity, in part owing to the reduced quantity of seeds (although there was almost always some amount present [47] (p. 315) [67] (p. 73–74) [83] (pp. 140–141) [176]). However, the confusion and association of seeds and tops continued until well into the nineteenth century: in 1854, the official *Pharmacopoeia Belgica Nova* defined *Cannabis* in its monograph as “an annual plant cultivated in our country. Its seeds are well known. Those imported from India contain Cannabine, a resin that is soluble in alcohol and ether” [310]. Around the same dates, in Germany, Freudenstein’s *De cannabis sativae usu ac viribus narcoticis* detailed resin extracted from the “leaves and seeds” as the preferred plant parts to use for the extraction of psychopharmacologically active resins [311] (pp. 17–18, 21–31), and in Valladolid, Spain, a pharmaceutical exhibition presented “*cañamones*” (hempseeds) as containing “a glutinous-resinous matter that, particularly the one cultivated in warmer countries like Persia, has the quality of inebriating and narcotising” [312] (p. 170).

The interplay between hempseeds and hemp tops, beyond the fact that it represented the consensus across Spanish society, may not have been entirely unrelated to the GI-related indications due to the association of hempseed with food practices—as addressed in the discussion of this article.

These two nondichotomical understandings of hemp and its plant parts are essential for understanding the historical discussions surrounding *Cannabis* in northeastern Spain and the broader Mediterranean region.

## Appendix C. From Sedenegi to Bangue: Hemp in Antique and Medieval Spain

Hemp preceded humans on the Iberian peninsula. Rull and colleagues [104] recently reviewed almost 60 items of archaeological pollen evidence, finding that:

*“The first scattered records of this pollen type date from the Middle and Upper Paleolithic […] and would have entered the [Iberian Peninsula] by maritime Mediterranean or terrestrial continental pathways, or both. A first burst of introductions, probably in a cultivated form, would have occurred during the Neolithic […] using similar paths. Human participation in these Neolithic introductions remains unclear but cannot be dismissed. A period of reduced Cannabis arrivals (mostly via maritime pathway) occurred between the Chalcolithic and the Roman Epoch […]. A second, likely anthropogenic, introduction acceleration took place in the Middle Ages […] using the Mediterranean and the continental pathways. Maximum cultivation and hemp retting activity was recorded during the Modern Ages”*

This appendix provides a brief historical background on the historical uses of hemp in medicine and pharmacy on the Iberian peninsula before the nineteenth century.

### Appendix C.1. Antiquity

During antiquity, a number of classical medical treatises from early Mediterranean natural philosophers mentioned hemp fruiting tops in some capacity, including sometimes in relation to digestion or stomach disorders. These include the Hippocratic corpus and works of Strabio, Dioscorides, Pliny the Elder, Pausanias, Mesue, Artemidorus, Athenaeus of Naucratis, and, notably, in many of Galen’s works [20,106] (pp. 145–147, 163–165, 470, 472) [110] (p. 27) [313]. The use of the plant by Roman cultures is well documented [314,315,316], and it could be hypothesised that their consumption of hemp products as psychoactive sweet meal at the end of banquets, described by Galen [111] (p. 68), [112] may not have been entirely foreign to the experienced digestive effect of the plant on the GI tract.

### Appendix C.2. Al-Andalus

On the Iberian Peninsula, the period known as “Al-Andalus” during the Middle Ages marked a turning point in hemp history. Although the plant was already present before the arrival of Arabo-Berberian cultures in the Peninsula, as was the case with other crops, Al-Andalus is associated with improvements and streamlining of hemp cultivation, apparently starting around the tenth century. Pipes used to smoke hemp tops appeared in the following century throughout the territory [103] (pp. 264–265), suggesting a certain level of knowledge, use, and diffusion by the time.

During the following centuries, notable medical, agricultural, terminological, ethical, theological, and legal discussions were taking place around hemp, its uses, and effects—analysed in the seminal works of Indalecio Lozano Cámara [113,114,115,116] and others [3] (pp. 18–20) [117,118,119,120,122] (p. 366).

Documents on the medical aspects of hemp also reappeared during that period, as classical Mediterranean authors were translated, complemented, and commented on, in a vanguardist medical scene [123]. These improved Andalusian texts served in large part as the basis for medieval Latin translations, which went on to form the textual corpus of large parts of early modern medicine across Europe [122] (pp. 110–24, 267–77, 360–69) [121,123] (p. 163) [124] (p. 31) [125] (pp. 293–301). Medical knowledge on hemp was also encompassed, although contemporary medical literature has often overlooked the relevance of the contributions of pan-Arabic scientists to European knowledge on the therapeutic properties of hemp [113].

The influence of Al-Andalus on hemp history and culture has been notable in the Catalonia region (besides a limited physical presence, from 717 to 801, compared with the southernmost parts of the peninsula). Present before Al-Andalus, hemp continued to be widely cultivated and used after the progressive Christian conquests of the peninsula (“*Reconquista*”), with permanence of the additional practical and scientific knowledge acquired during that period.

### Appendix C.3. Reconquista

It seems that all parts of hemp plants (see also Appendix B) continued to be used throughout the Iberian Peninsula after the *Reconquista* (some authors assert that hemp then became associated with paganism, banned, or repressed, Ref. [10], but little primary evidence supports this). In 1555, Miguel Juan Pascual dedicated a determined defence of hemp cultivation as an answer to “[his] highest Inquisitor authorities, of whom [he is] the family doctor” [313], documenting extensive cultivation in the northeastern coast of the peninsula between València and Barcelona: “there are many cities and villages in Hispania that are very salubrious, despite having in great abundance […] cultivations of cannabis”.

Both its seeds [317] and its “leaves, that the Arabs call al-axixha” [318] (p. 26) appear to have remained part of Spanish alimentation, at least in the countryside. The consumption of hemp tops for their psychopharmacological activity, smoked or masticated, is documented after Al-Andalus. Various excavations have uncovered remains of hemp tops in smoking pipes across Spain [319] (p. 156), including hemp-smoking pipes in the outskirts of Barcelona dated between the eleventh and thirteenth centuries, leading archaeologists to “confirm the use of hemp as a smokery in the medieval Christian world.” [103] (p. 266).

In northeastern Spain, another custom that seems to have endured until the nineteenth century [126,127] (p. 87) was the chewing of hemp tops. The *Biblioteca de Catalunya* conserves a manuscript containing a recipe for “Powdered Tobacco from Brazil with strong haschisch” from the early eighteenth century [128].

Medicinally, there is also evidence of continued use, with the circulation of classical treatises and the publication of new ones. In 1489, the Catalan translation of Guy de Chauliac’s Antidotary was published, which recommended the use of topical hemp for treating chest pain [320,321]. In relation to GIDD, we find hempseeds in another Catalan formulary containing a “remedy against stomach ache” [322] (pp. 48–49) attributed to Joaquim Bahí Puig (1728–1807) [323] (pp. 248) in a pocket-sized book reprinted several times until the late nineteenth century [324].

### Appendix C.4. Sedenegi Quid?

Parallelling this continued presence and use, some texts suggest a certain discontinuity or disconnect between early medical and pharmaceutical knowledge and the development of hemp medicines in modern Spain. In 1603, the early pharmacopoeia of València’s College of Apothecaries reproduced the classical recipe from Mesue [325] (p. 43) [326] of the “*Trochisci de terra Sigillata*” listing among the ingredients two ounces of “sedenegi”. A footnote attached, titled “*Sedenegi quid*” (“What is sedenegi?”), adding:

*“note that the Arabic expression Sedenegi […] signifies seeds of Canabis, although some believe it is fumitory seed [Fumaria officinalis], while other authors pretend that word means hematite stone.”*
[327] (p. 283)

An Arabized noun derived from Persian *šāh dānag* (“the king of grains”/“the sultan of seeds”) [115] (pp. 121n22, 184, 209) [328] (p. 293), the word “*šedenegi*” (“*šahdānaŷ*”) was a key term for consumable hemp fruiting tops during Al-Andalus [114] (pp. 153–155) [116] (pp. 152–155) (as evoked earlier, it is challenging to ascertain whether *šedenegi* referred to the entire seeded tops with leaves and pericarps bearing phytocannabinoid-containing trichomes, or to seed kernels separated from vegetable parts).

This uncertainty of seventeenth century apothecaries about the meaning of the *šedenegi* could be indicative of a certain degree of knowledge loss at that time.

The confusion may have originated in Simon of Genoa’s thirteenth century medical dictionary, in which hematite stone was called “*sabdenegū*” next to cannabis defined as “*sadenegū*”/“*ꝓscehedenigi*.” [329] (pp. 267–268); some pages later, the word “*sceheterigi*” was given for fumitory and, just below, “*scehedenigi*” meant cannabis [329] (p. 275). At the end of the fifteenth century, Nebrija’s influential dictionary, reedited for centuries, contained “cannabis” and “cañamo ierva” (hemp herb) but not “sedenegi” [330]. The word seems to have prevailed longer in Mediterranean coastal areas, appearing in Barcelona’s 1511 *Concordia* (often considered to be among the first official pharmacopoeias in Europe) and in other Catalan texts of the sixteenth and seventeenth centuries (although it is unclear whether it was used to designate hematite, cannabis, or both) [331] (pp. 234, 518) [332] (p. 20). Outside Spain, not all authors lost the meaning of the word; some even discussed at great length the terminological confusions surrounding it [333] (pp. 85–90). The most recent, accurate use of the term was found in a 1680 French translation of Mattioli’s comments on Dioscorides’ *Materia Medica* (Lib. III, Ch. CXLVIII) conserved in Barcelona University archives, where multilingual synonyms were provided:

*“Cannabis. French: Chanvre, or Chenève. Arab: Scehedenegi & Canab. Italian: Canape. German: Zamer hanff. Spanish: Canthamo”*
[334] (pp. 355–356)

Today, the term is often overlooked [95], or is not associated with *Cannabis* [335,336] (pp. 278, 451): a historical naming confusion resulting in a loss of medical memory, previously observed with other medicinal plants such as *Valeriana officinalis* [130].

As hemp terminology inherited from Al-Andalus faded away (with the notable exception of “haschisch”), new words appeared in Spain and Portugal with early explorers and commentators of *Materia Medica* “telling the things” from Africa, Asia, and America [131] (pp. 62–72) [132] (pp. 73–85).

### Appendix C.5. The “New World”

In the sixteenth century, Portuguese explorer Diego Garcia d’Orta described in his *Colóquios* (translated into many European languages) the plant “bangue” (probably derived from the Hindi term *bhang* or Swahili *bangi*, both meaning hemp [15] (p. 136)) compared with his native “linho alcanave” (a term derived from *linum*, Latin for “flax”, and *al-qannab*, “the cannabis” in Al-Andalus dialect) [337] (pp. 75–78). The same occurred some decades later when Cristobal Acosta stated in his *Treatise on drugs and medicines of Eastern Indies* that “Bangue is a plant similar to hemp, the Canabis of the Latins, as Dioscorides said…” [338] (p. 359). Nicolás Monardes (a slave trader from Sevilla, and best-selling medical author throughout sixteenth- and early seventeenth-century Europe, who had never set foot outside of the peninsula [132] (pp. 74–77)) relayed to an even broader European readership the recent findings about “bangue”, its similarity to common hemp, and its potential medical applications.

Together, these Iberian authors imparted what could be called a *new wave* of interest in hemp from the medical standpoint in Spain. Among their readers, outside of the peninsula, we find Lyon’s *Hôtel-Dieu* doctor Jacques Daléchamps—whose 1653 plant treatise was influential in the later development of the European hemp discussion [19] (pp. 35–36, 236n68)—or Swedish botanist Carl von Linnaeus [131] (p. 65) who would later theorise hemp/bangue/cannabis as a unique, single genus and species [302].

As Roig-Traver noted [3], the preexisting familiarity of Spanish health professionals with the hemp plant, its medical uses, and its psychopharmacological characteristics, may have facilitated later acceptance of hemp as a reliable medical ingredient in the nineteenth century.

## Appendix D. Background Information on Parke-Davis and Dausse—The Main Wholesale Suppliers of Hemp Extract to Spanish Pharmacies During the Period Studied

The first homogenisation methods were developed by large pharmaceutical companies with important presence worldwide, including Spain, in particular:

- Parke, Davis & Co. (Detroit/London);
- Dausse Aîné/Boulanger-Dausse (Paris);
- Burroughs-Wellcome (London), to a minor extent.

These companies not only played pivotal roles in the standardisation of medicinal hemp products but also in scaling-up production, distribution, and consequently access, on a global scale. These important suppliers (particularly Dausse and Parke-Davis, reviewed below) and their importance in the medicinal hemp trade, are not well documented in the contemporary literature.

### Appendix D.1. Dausse

The laboratories Dausse (Dausse-Aîné, Boulanger-Dausse, Duboë-Dausse, later Synthélabo, today part of Sanofi) were founded in Paris in 1834. Dausse carved out a niche as a pioneer in the mass production of herbal extracts, acquiring a notable presence in the pharmaceutical industry Europe-wide. Dausse’s soft and liquid extracts are mentioned in Spain for the first time in 1875 [54] (pp. 248–255), with imports starting in 1876 and a first Spanish catalogue a decade later [175] (pp. 72,97), with a documented presence at least until 1928. In order to ensure the homogeneity and stability of its hemp extracts amidst the prevailing uncertainty on its active principles, but also variables such as the presence of seeds in hemp tops resulting in “some variation in the amount of extractive obtained” [176], the company had developed its own physiological standardisation method for hemp by 1907 [177] (pp. 317–318) [178] (pp. 42–46) [179] (p. 931). In addition to tinctures and numerous formulae, Dausse proposed the nine types of physiologically standardised extracts (90º alcohol, vacuum evaporated at 40 °C, except where noted otherwise):

- “Haschischine (i.e., hydroalcoholic soft extract of “Indian hemp”);
- Hydro-alcoholic soft extract of “indigenous hemp” (Dausse cultivated hectares of common French hemp, near Paris, to produce this extract [175] (pp. 132, 214) [178] (p. 53));
- Hydroalcoholic soft extract of “Indian hemp” (using 60º alcohol);
- Ethereal soft extract of “Indian hemp”;
- Hydroalcoholic dry extract of “Indian hemp”;
- Fluid extract of Indian hemp (90º alcohol for equal weight of plant material);
- Fluid extract of haschischine (90º alcohol with ⅓rd of the weight in haschischine;
- Fatty extract of Indian hemp in cocoa butter;
- Aqueous extract of Indian hemp.

### Appendix D.2. Parke-Davis

Another key company was Parke, Davis & Co. (Parke-Davis; today part of Pfizer), founded in 1862 in Detroit. It emerged as the first pharmaceutical manufacturer in human history to achieve a worldwide presence, in the early twentieth century [180]. Among other medicines, the company provided particularly noteworthy contributions to modern botanical and pharmaceutical knowledge on hemp.

The fluid extract of hemp was already present on the first catalogue of the company [181]. The plant remained one of Parke-Davis’ research and market foci for decades, until a notorious international dispute between the company and the government of Egypt, in the early 1930s [182]. Like Dausse, Parke-Davis cultivated, in Detroit, its own fields of traditional hemp varieties bioprospected in Central America [176,183].

Parke-Davis invested significantly in science at their emblematic Detroit Research Laboratories [180] (p. 80) [184] (p. 70). Early on, they “entered upon a systematic investigation of the *Cannabis sativa*” resulting in the development by 1896 of a way to standardise their fluid, solid, and powdered extracts of hemp and other plants claimed to be the “first line of physiologically assayed and standardised extracts ever known to pharmacy” [1] (pp. 40–52). Their homogenisation method (the “dog ataxia test” [185] (pp. 16–17)), was improved by Herbert C. Hamilton, the company’s lead cannabis researcher [98] (pp. 6, 447), who shared it in medical journals during the first decade of the twentieth century, thus encouraging its replication and popularisation on a global scale. In Barcelona, the Faculty of Pharmacy’s textbook *Medicamenta* (reprinted several times from the 1920s to the 1960s) participated in the diffusion of Parke-Davis’ method:

*“Adult dogs weighing less than 13 kg which have been found to be sensitive to Indian hemp should be used for titration; […]. The extract should be taken by mouth in gelatin capsules and the results should be observed one hour later.”*
[186] (p. 89)

What could anachronistically be described as early *open science* by Hamilton and Parke-Davis certainly accelerated or facilitated the development of other hemp homogenisation strategies through physiological methods, such as that of Dausse (as we have just seen) or the British laboratories of Burroughs, Wellcome & Co. in 1913 [187] (p. 20), another company importing hemp extracts into Spain (today part of GSK plc).

Parke-Davis had been present in Europe since 1888, and in Barcelona since 1890. The first Spain-specific catalogue is documented in 1902: on its first page, it presented its “treatment and physiological assay of fluid extracts” citing “Indian hemp” as an example (Figure A3a).

The company proposed a total of 55 hemp-containing medicines including at least: [1] (pp. 45–47) 15 pills (3 of pure hemp extract; Figure A3c), 11 tablet triturates (4 pure), 7 water-soluble compressed tablets, 12 chocolate-coated tablets (3 of pure hemp extract or tincture), 5 liquid preparations, as well as packs of 1 ounce of hemp tops “compressed for retailing”, two types of tinctures, and the following extracts:

- Fluid extract “No. 106” (Figure A3b; sometimes called “Normal liquid”);
- Solid extract “No. 35”;
- Powdered extract “No. 16”, at one fourth of the strength of the former.

The last Spanish Parke-Davis catalogue which could be consulted, from 1931, still contained Fluid Extract of Indian Hemp No. 106.

Interestingly, Parke-Davis did not mention GIDD indications as often as Spanish sources did. Reflecting in part the findings of Mikuriya that we discussed in Section 2, the documentation consulted from the company showed a comparatively lower interest in GI and stomachal indications, when compared to migraine/headaches and insomnia, in particular [1] (p. 141). Among the scarce mentions, however, Parke-Davis did claim in 1894 that “the peculiar sedative influence exerted by Indian cannabis within the intestinal tract is almost magical” [188] (p. 21) [189] (p. 39). The recommendation appeared again in following years, until at least 1937 [1] (p. 145) [190] (p. XXXVII-XL).

The primary documentation consulted in Spain did not yield any mention of pharmaceutical companies such as Eli Lilly (often cited in popular cannabis history texts) supplying cannabis to Spanish pharmacists. In contrast, it is surprising to find so few mentions of Dausse or Parke-Davis in the secondary literature on Spanish and French “medical cannabis history” when compared to the general importance of these companies at the time.
